# Effect of low-frequency noise exposure on cognitive function: a systematic review and meta-analysis

**DOI:** 10.1186/s12889-023-17593-5

**Published:** 2024-01-09

**Authors:** Peng Liang, Jiangjing Li, Zenglei Li, Jing Wei, Jing Li, Shenghao Zhang, Shenglong Xu, Zhaohui Liu, Jin Wang

**Affiliations:** 1 Department of Rehabilitative Physioltherapy, The Second Affiliated Hospital of Air Force Medical University, Xi’an, 710038 China; 2 Hospital of No. 95007 Unit of PLA, Guangzhou, 510410 China; 3 Department of Anesthesiology, The Second Affiliated Hospital of Air Force Medical University, Xi’an, 710038 China; 4 Department of Radiation Medical Protection, Ministry of Education Key Lab of Hazard Assessment and Control in Special Operational Environment, School of Military Preventive Medicine, Fourth Military Medical University, Xi’an, 710068 China; 5 Department of Neurosurgery, the 940th Hospital of PLA Joint Logistics Support Force, Lanzhou, 730050 China; 6 Department of Orthopaedics, The Second Affiliated Hospital of Air Force Medical University, Xi’an, 710038 China

**Keywords:** Low-frequency noise, Noise pollution, Cognition, Higher-order functions, Meta-analysis, Systematic review

## Abstract

**Background:**

Low-frequency noise may cause changes in cognitive function. However, there is no established consensus on the effect of low-frequency noise on cognitive function. Therefore, this systematic review and meta-analysis aimed to explore the relationship between low-frequency noise exposure and cognitive function.

**Methods:**

We conducted a systematic review and identified original studies written in English on low-frequency noise and cognition published before December 2022 using the PsycINFO, PubMed, Medline, and Web of Science databases. The risk of bias was evaluated according to established guidelines. A random-effects meta-analysis was performed where appropriate. To explore the association between low-frequency noise exposure and cognitive function, we reviewed eight relevant studies. These studies covered cognitive functions grouped into four domains: attention, executive function, memory, and higher-order cognitive functions. The data extraction process was followed by a random-effects meta-analysis for each domain, which allowed us to quantify the overall effect.

**Results:**

Our analysis of the selected studies suggested that interventions involving low-frequency noise only had a negative impact on higher-order cognitive functions (Z = 2.42, *p* = 0.02), with a standardized mean difference of -0.37 (95% confidence interval: -0.67, -0.07). A moderate level of heterogeneity was observed among studies (*p* = 0.24, I^2^ = 29%, Tau^2^ = 0.03).

**Conclusions:**

Our study findings suggest that low-frequency noise can negatively impact higher-order cognitive functions, such as logical reasoning, mathematical calculation, and data processing. Therefore, it becomes important to consider the potential negative consequences of low-frequency noise in everyday situations, and proactive measures should be taken to address this issue and mitigate the associated potential adverse outcomes.

**Supplementary Information:**

The online version contains supplementary material available at 10.1186/s12889-023-17593-5.

## Background

Noise is a well-known environmental stressor that can negatively affect physiological, psychological, and behavioral processes [1, 2], making noise pollution a significant global public health concern. In the United States alone, over a quarter of the workers have been negatively impacted by noise. Furthermore, in its 2020 environmental noise report, the European Environment Agency stated that more than 22 million individuals were adversely affected by noise [3]. Low-frequency noise (LFN) refers to a type of environmental noise characterized by sound waves below 200 Hz. It is considered a distinctive environmental noise issue that affects many households [4]. The sources of LFN can be both natural, including wind, thunder, lightning, waves, earthquakes, and volcanic eruptions, and artificial, including life-related noise (e.g., air conditioning, power distribution equipment, elevators, and fans), traffic noise (e.g., railways and aviation), industrial enterprise noise (e.g., substations, wind farms, and converter stations), and construction noise (e.g., piling and decoration). As LFN becomes more prominent, its effects on humans intensify [5]. Studies have demonstrated the negative effect of LFN on the circulatory, endocrine, and nervous systems of the human body as well as on learning and social behavior [6, 7]. The impact of LFN can often be masked by medium and high-frequency noises, leading to people being less aware of the effects of LFN. Moreover, environmental noise measurements typically involve A-weighting, which significantly attenuates low-frequency components, potentially resulting in individuals being strongly disturbed by LFN, even when measurement values are within the standard limits [8]. The World Health Organization has also emphasized the detrimental effects of LFN for the first time in their *Guidelines for Community Noise *[9]. These recommendations about LFN were developed in the *World Health Organization Night Noise Guidelines for Europe *[10], and the noise guidelines are constantly improving over time [11, 12]. According to another review, it was reported that about 10% of people lived near infrasound or LFN sources, causing diseases of the central nervous system. LFN in the daily environment constitutes a problem that requires more research attention [13].

Cognition refers to the abilities related to the acquisition, processing, utilization, and understanding of information, which involves various functions, such as attention, learning, memory, execution, reasoning, and calculation [14]. Cognitive ability is not only a measure of learning capacity but also an important indicator of physical and mental health. Studies have demonstrated that environmental noise can negatively impact cognitive function [15, 16]. The potential causes for these negative impacts include distraction, reduced sleep quality, reduced speech perception ability, elevated psychological stress, discomfort, and learned helplessness [17–20]. Through analysis of brain structure and organization, it was theorized that this change in cognitive function may be related to gray matter decline in cognition-related brain regions such as the cerebellum and angular gyrus [13] and may also be related to the Ca^2+^ mediated apoptosis pathway in the hippocampal neurons [21].

Previous studies have examined the association between environmental noise and cognitive function, although most studies have included traffic noise rather than LFN as an independent exposure factor for their analysis and research [15, 16]. Some scholars propose that the adverse effects of noise can be attributed to the LFN component, with the rattling or vibrating aspect of LFN potentiating these negative effects [22]. However, research on LFN in occupational environments remains limited, and its negative impacts have not been widely recognized to date [23].

We proposed the question of whether LFN exposure increases the risk of cognitive impairment in humans compared to less LFN exposure. Therefore, this literature review considered LFN as an individual exposure factor. We aimed to systematically analyze and assess the impact of LFN on cognitive function by assessing the risk of bias in the studies and highlighting the primary limitations of existing research. Collectively, these findings can provide a useful theoretical foundation for the development of effective noise protection policies.

## Methods

### Protocol and registration

This systematic review was conducted according to the PRISMA standards [24] (Additional file 1), and the protocol was pre-registered in the PROSPERO database (ID = CRD42022384598) on December 27, 2022.

### Eligibility and inclusion and exclusion criteria

This systematic review was performed in strict accordance with the Population, Exposure, Outcome framework [24]. The PECOS (participants, exposure, control/comparison, outcomes, and study design) statement is described in Table 1. For the literature review, only original research articles were included, whereas review papers, conference records, abstracts, editorials, reports, letters, notes, chapters, books, and theses were excluded. No restrictions were imposed on the publication year or geographic area; however, given the need for accurate understanding of the content and unavailability of translation sources, we only included research papers written in English. We also established specific inclusion criteria pertaining to the exposure method and outcome of intervention factors as follows:

- 1) The presence of clear acoustic calibration instructions for noise exposure as an intervention condition, such as the spectral analysis of noise, and the primary component of noise exposure should be explicitly stated as the LFN component.
- 2) The reporting of cognitive function domains, including performance on neurocognitive tasks, academic skills, overall IQ, measurements of neurodevelopment, and cognitive decline.

**Table 1:** The statement of PECOS

Category	Statement
Participants	Human populations across the lifespan
Exposure	Low frequency noise (exposure to noise in a lab to measure concurrent effects on cognition)
Control/Comparison	Persons less exposed
Outcomes	Non-pathological cognitive abilities
Study Design	Experimental study designs

### Information sources

The literature was sourced from the PsycINFO, PubMed, Medline, and Web of Science databases. Using keywords centered around “low-frequency noise” and “cognition,” searches were conducted to identify all published articles relevant to both concepts. The search period spanned from the earliest available date in each database until December 16, 2022. The complete search strategy for each database can be found in Additional file 2.

### Study selection

The selected studies bibliography was created using the NoteExpress reference management software. After consolidating the literature and removing duplicates, two authors independently reviewed the literature based on the inclusion criteria. Initial screening involved reviewing the titles and abstracts of all articles, followed by screening the full text. Any discrepancies or uncertainties regarding eligibility or information extracted were resolved through discussion or consultation with other authors. If more detailed information was required from the original article, the authors were contacted for a joint decision on the inclusion or exclusion of the article.

### Data collection process

Data were independently collected by the first author using a standardized data extraction table, primarily including data, such as article’s authors, publication year, study field, research design, noise type and assessment, results, measurements, adjustments, and effect sizes reported. A second researcher reviewed all the extracted data, and discrepancies were resolved through discussion. All reviewers and data extractors have received unified training and learned about the use of the Revised Cochrane risk of bias tool for randomized trials (RoB 2) [25]. Missing data were obtained by contacting the corresponding authors through email. When the data could not be used directly for analysis, corresponding formulas were applied for data conversion, and subgroup data were reasonably combined. For example, in the study of Belojević G et al. [26], participants were classified based on noise sensitivity, but in other articles, study cohorts were randomly selected from the entire population. Therefore, we merged the three subgroups. First, we merged the two subgroups with medium and low sensitivity to noise, and then we merged the obtained data with the subgroup with high sensitivity to noise to obtain the effective data. The specific extraction and conversion of raw data can be found in Additional file 3.

### Data characteristics

Data were extracted from all included articles based on the following four characteristics: 1) intervention and control measures, including the intensity and type of LFN and whether the control group was less exposed; 2) basic subject information, including sample size, age range, and gender differences; 3) experimental design methods, including randomization methods, blinding, within/between-subject designs, and other methods; 4) extraction of continuous variable data, including the mean, standard deviation, and sample size of each group. If the original text did not provide corresponding means or standard deviations, other convertible statistical data, including standard errors, 95% confidence intervals (CIs), and t- or *p*-values, were extracted.

### Risk of bias and quality of evidence assessment

Two authors independently assessed the risk of bias in the studies using the RoB 2 for randomized trials [27]. This tool assessed potential threats to the internal validity of randomized controlled trials across six domains of bias: randomization process, deviations from the intended interventions, missing outcome data, measurement of outcomes, selection of the reported result, and overall bias. The possible risk-of-bias judgments for each domain comprised a low risk of bias, some concerns regarding bias, and a high risk of bias [28, 29]. The individual and overall bias risks were strictly evaluated based on the guidelines provided by RoB 2 [30]. Any discrepancies were resolved through discussion or consultation with a third author. We further evaluated the overall quality of evidence and strength of evidence assessments for each outcome using the Grading of Recommendations Assessment, Development, and Evaluation (GRADE) guidelines. The starting level of the quality of evidence was determined according to the study design. This initial level was then increased or decreased considering several factors. Factors that lower the confidence of evidence were: risk of bias, inconsistency of results, indirectness of evidence, imprecision, publication bias, and number of studies. In contrast, a dose–response gradient, a large magnitude of effect, and confounding that underestimate the associations can increase confidence [31].

### Summary and synthesis of results and meta-analysis

Due to systematic or random errors, the studies included in the meta-analysis may produce results that deviate from the real population parameters. The purpose of integrating these results into the meta-analysis is to generate estimates that are closer to the real population parameters by minimizing these errors in the study. Meta analysis models are mainly divided into conventional fixed effects and random effects models [32], among which there are also inverse variations, quality effects, and random effects estimators that are used to reduce these errors as much as possible, but there is still a controversy about the most effective estimator [33]. If heterogeneity is expected between relevant research results, a random effects model is usually preferred for analysis [34]. This model handles heterogeneity between data by increasing the weight of large sample data and reducing the weight of large sample data. Although this method carries certain risks, considering the variations in noise intervention, outcome measurement, and target populations across the included studies, a random effects model was still adopted to obtain more conservative results [35, 36]. Using RevMan software, the standardized mean difference (SMD), 95% CI, and *p*-value for different cognitive tasks were calculated. The Q statistic was used to examine the heterogeneity of study results, with a *p*-value of < 0.05 indicating heterogeneity across studies. Additionally, the I^2^ statistic was utilized to quantify the impact of heterogeneity. Low, moderate, and high heterogeneity were represented by an I^2^ ≤ 25%, 25%–75%, and ≥ 75%, respectively. Heterogeneity was generally considered acceptable if I^2^ did not exceed 50% [37, 38].

## Results

### Study selection

**Fig. 1:**
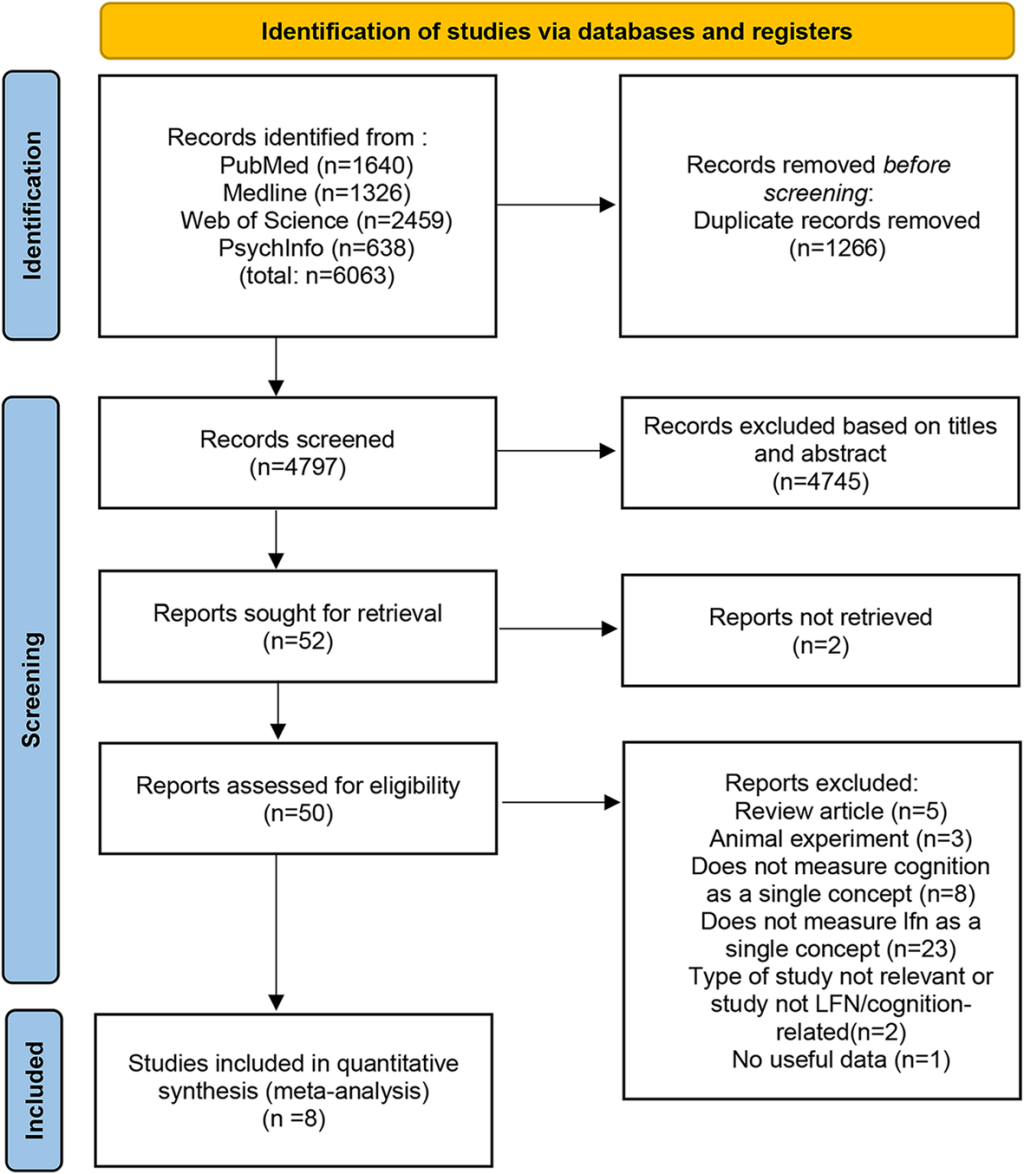
PRISMA-style flowchart of the study selection process

This study included a total of eight original randomized control studies from five different countries, with three articles from Sweden and two from Iran. The specific selection process is shown in Fig. 1. The exclusion reasons for 42 full-text articles can be found in Additional file 4.

### Study characteristics

In this study, cognitive function assessment was further classified into four domains: attention, executive function, memory, and higher-order cognitive functions [38]. Detailed information on the classification of functions in each included study as well as in-depth descriptions of LFN are outlined in Table 2.

**Table 2:** Descriptive characteristics of studies

Reference	Author	Year	Sample	Details of Intervention	Type of LFN	Cognitive Domain	Cognitive Tasks	Details of Tasks		Std.Mean Difference (95%CI)
Hearing, communication and cognition in low-frequency noise from armoured vehicles	Nakashima, Ann; Abel, Sharon; Duncan, Matthew; Smith, David	2007	*n* = 36; age range: 18–55	The subjects were tested with the ears unoccluded and while wearing a David Clark headset (H10-76XL) in both passive and ANR modes (referred to as “ANR Off” and “ANR On,” respectively)	The LFN (hereafter referred to as vehicle noise) was recorded near the ear of the crew commander while standing with his upper body through the hatch of a LAV III. The vehicle was traveling on a highway at approximately 80 km/h	Attentional functioning	Four-choice serial reaction time (SRT)	For the SRT task, one of four probe letters was presented on the screen (P, G, L and S), and the subject pressed the corresponding response button as quickly and accurately as possible		1.09 (0.22,1.96)
						Executive functioning	Mental addition (MA)	The MA task required the subjects to add a random sequence of eight numbers (between 1 and 16) that were presented one by one on the computer screen		-0.05 (-0.85,0.75)
						Memory	Detection of repeated numbers (DRN)	In the DRN task, a series of three-digit numbers were flashed on the screen at a rate of 2/s. The subjects pressed a button when a three-digit number was repeated		0.11 (-0.69,0.91)
						Higher-order functions	A logical reasoning task (LRT)	For the LRT, a pair of letters was shown at the top of the screen (A B or B A) and a statement showing the spatial arrangement of letters was shown below, e.g., “B does not follow A.” The subjects responded by indicating whether the statement was “true” or “false.”		-0.79 (-1.62,0.05)
A longitudinal, randomized experimental pilot study to investigate the effects of airborne infrasound on human mental health, cognition, and brain structure	Ascone, L; Kling, C; Wieczorek, J; Koch, C; K¨^1^hn, S	2021	*n* = 38; age mean: 19.76	Six sources were assembled in total, three fully functional sources for the verum group and three dummy sources for the placebo group. The sources were designed to be installed by the setup team in the participants bedroom and then to work for several weeks autonomously and reliably. The main components were a frequency generator, producing a 6 Hz sinusoidal tone, an amplifier,a loudspeaker, and a 26 m flexible tube attached to the speake	The IS sources were configured in such a way that they would emit a steady SPL between 80 and 90 dB (6 Hz frequency) for eight hours during the participant’s self-reported habitual sleep (bed) time interval	Attentional functioning	Alertness	Concerning alertness, tonic and phasic alertness can be differentiated. Tonic arousal is a good proxy for wakefulness, where the participant is in a state of willfully maintaining his or her attention and accordingly responds quickly to an upcoming event		-0.39 (-1.05,0.27)
						Executive functioning	Go-nogo	Concerning alertness, tonic and phasic alertness can be differentiated. Tonic arousal is a good proxy for wakefulness, where the participant is in a state of willfully maintaining his or her attention and accordingly responds quickly to an upcoming event		-0.57 (-1.24,0.09)
						Memory	Sustained attention	Sustained attention refers to the capacity to concentrate on a routine task for a longer period of time (15 min in the case of the test)		0.01 (-0.63,0.65)
						Higher-order functions	Incompatibility		Incompatibility effects occur in conflict situations, where divergent information generates a conflict in reaction tendencies	-0.52 (-1.18,0.14)
Effects of noise on mental performance with regard to subjective noise sensitivity	Belojević, G; Ohrström, E; Rylander, R	1992	*n* = 45; age range: 18–32	Three different acoustic conditions were used in the experiments Background laboratory sound of 30 d B(A) Leq (measured at the subjects' seats) constituted a quiet ambient condition, while the recorded motorway noise, continuous and variable, was emitted at two levels: moderate[Leq = 55 d B(A), L 99 = 48.2 d B(A), L = 62 9 d B(A)l and high lLeq = 75 d B(A), L 99 = 68.2 d B(A), L 1 = 82 1 d B(A)]	The spectral analysis of noise showed a dominance of lower frequency components (40–400 Hz) due to the high proportion of heavy vehicles in motorway traffic	Executive functioning	The Mental Arithmetic task (MA)	The Mental Arithmetic task (MA), a test of parallel processing. A set of 15 tasks invovled mental division of two-digit numbers by 6, 7, 8, or 9, with a two-decimal result The efficiency and the depth of mental processing were estimated by the number of correct whole numbers in the results (shallow), one-decimal results (intermediate), a total results (deep), and by the time spent to complete the task		0.21 (-0.20,0.63)
						Memory	The Short-Term Memory task (STM)	On a computer monitor, 25 series of seven consonants, appearing consecutively with a few seconds' pause, were shown to the subjects In addition, 12 words of different semantic categories appeared occasionally during the projection of letters Consonants had to be recalled by order of appearance and registered on a form after each series The subjects were advised to memorize the letters in groups of 3 + 4 in order to avoid the influence of individual strategies in mental performance The words had to be memorized and written only upon completion of the entire test, without attention to their order The performance was measured by the number of errors and by the number of words remembered in the groups of the same semantic category (clustered) The test lasted 25 min		-0.12 (-0.54,0.29)
Effects of road traffic noise and irrelevant speech on children's reading and mathematical performance	Ljung, R; Sörqvist, P; Hygge, S	2009	*n* = 187; age range: 12–13	In the noise conditions, digital recordings of road traffic noise or irrelevant speech were played back through loudspeakers at the front of the classroom. The equivalent sound level (Leq) in the noise conditions was set to 66 dBA 2 m in front of the loudspeakers. The road traffic noise recording was made up of a background of continuous road traffic noise (~ 62 dBA) with superimposed segments of trucks passing by. The peaks in the superimposed segments were at 78 dBA	The dominant frequency range for the road traffic noise (100–300 Hz) was lower than that for the irrelevant speech (500–1500 Hz)	Executive functioning	Reading Test	The test consisted of a four-page story. With regular intervals there was a critical choice point. At these points, three words were presented. Each word was grammatically correct in the phrase in which they occurred, but two of them were wrong in the context. The task was to underline the word that was correct in the context		-0.21 (-0.20,0.63)
						Higher-order functions	Basic Mathematics	This test consisted of arithmetical and geometrical problems. The arithmetical tasks were division (three problems) and multiplication (three problems). The geometrical problems were naming points in a coordinate system (two problems), understanding of the relationship between fractional expressions and areas of figures (four problems), understanding of the relationship between distance and numerical expressions (two problems), and measuring of distances (two problems)		-0.49 (-0.86,0.12)
Low frequency noise "pollution" interferes with performance	Persson, Waye K; Bengtsson, J; Kjellberg, A; Benton, S	2001	*n* = 32; age mean: 23.3	The sound was produced by four loudspeakers, hidden behind curtains and placed in each comer of the room To amplify the low frequency noise, there was a subwoofer (ace-bass B2-50) which can reproduce frequencies down to 20 Hz. The background noise from the test chamber ventilation was less than 22 dBA, and the sound pressure levels for frequencies below 160 Hz were below the threshold of normal hearing [ISO389-7:1996]	To obtain the low frequency noise, sound pressure levels in the frequency region of 31.5 to 125 Hz were increased using a digital sound processor system	Memory	Short-time Memory Task	A set of numbers, e.g. 1 2 5 4, was shown on the computer screen. This set was followed by one number, e.g. 7. The subject was to respond, by yes or no, to whether that number was also present among the set of numbers shown earlier. The total response time and total number of correct and false answers were recorded		-0.26 (-0.38,0.20)
The Effect of Road Traffic Noise on Reaction Time	Alimohammadi, I; Zokaei, M; Sandrock, S	2015	*n* = 80; college students	At first stage the participants were asked to do the above mentioned RT test in the acoustic room in quiet condition (with background noise of 32.9 dBA) equipped with universal panel of Vienna test system. In second stage case group participants were exposed to traffic noise levels with 72.9 dBA for duration of two hours and then they did the RT test. In this stage, control group participants had been sat in acoustic room for two hours without noise and then they did RT test	In this experimental study, participants were exposed to road traffic noise with level of 72.9 dBA recorded and measured at ninety points in a central parts (that have often heavy traffic) of Tehran, Iran in 2012(31.5–500 Hz). Noise was measured at A– frequency weighting and fast time weighting. During the traffic noise measurement, the traffic noise was recorded by a high quality voice recorder	Attentional functioning	Reaction Time (RT)	Reaction time was measured by RT test from Vienna Test System. Test form S5 was used in this study. This test form assess reaction time (split into reaction and motor time) in response to simple and complex visual or acoustic signals		0.46 (-0.02,0.90)
						Executive functioning	Movement Time	The mechanical response movement consists either of two visual stimuli (yellow and red lights) or a visual and an acoustic stimulus (yellow light and tone at 2000 Hz). The respondents are instructed to respond less than 2 s otherwise the alternative signals are appeared. The time from the presentation stimuli on the monitor to taking index finger from golden button was considered as movement time		-0.06 (-0.50,0.38)
The effects of low frequency noise on mental performance and annoyance	Alimohammadi, Iraj; Sandrock, Stephan; Gohari, Mahmoud Reza	2013	*n* = 90; age mean: 23.46	After 30 min, subjects performed Stroop and Cognitrone tests again. During performing tests, there was LFN as well. In other words, the exposure duration to LFN was 30 min plus test performance time. For reducing recall bias, half of the subjects were exposed to 50 dBA at first, and the others were exposed to 70 dBA. At the beginning of each test, sound pressure level was measured near the head of the subject in octave band frequency by B&K (model 2238)	LFN in 50 and 70 dBAwas produced inside the acoustic room by Cool Edit Pro. 2.1. Some of the main sources LFN are computers, printers, and air conditioner systems, usually known as quiet devices. In many administrative offices, the Leq = 50 dB noise can be considered as quiet conditions Meanwhile, based on the authors experience, the noise level in the relatively crowded offices is around Leq = 70 dB	Attentional functioning	Cognitrone Test	Cognitrone test was used for evaluating attention and concentration via identical comparison of figures. In other words, Cognitrone test was applied to assess the attention and concentration through comparison of figures with regards to their congruence		-1.04 (-1.35, -0.73)
						Executive functioning	Stroop Test	Stroop Interference Test is a sensorimotor speed test registering speed performance when reading words and naming colors and the speed performance under color–word interference conditions		-0.03 (-0.32,0.26)
The impact of low-frequency noise on human mental performance	Pawlaczyk-Luszczyniska, Malgorzata; Dudarewicz, Adam; Waszkowska, Malgorzata; Szymczak, Wieslaw; Sliwinska-Kowalska, Mariola	2005	*n* = 96; age range: 19–27	Experiment was performed in a special chamber for psychological tests (6.8 m^2^) furnished as an office environment. The preselected subjects, categorized in terms of individual sensitivity to LFN, performed standardized psychological tests during exposure to LFN or reference noise at the same equivalent-continuous A-weighted sound pressure level (SPL) of approx. 50 dB. Each subject carried out tasks once at randomly-assigned exposure conditions	The noise was generated by a set of loudspeakers placed in the corners of the room. Low frequency noise simulated noise occurring in the industrial control rooms. The reference noise was the broadband noise of a predominantly flat frequency character, without dominant low frequency components	Attentional functioning	The Continuous Attention Test (DAUF)	For thirty minutes, rows of triangles are presented on screen under time-critical conditions; the tips of the individual triangles can point either up or down. When a previously determined amount of triangles points down, the subject has to press the reaction button		-0.12 (-0.52,0.28)
						Executive functioning	The Stroop Color-Word Test	It is based on the assumption that reading speed of a color-word is slower, if the word is written in a differently colored font. There is always a delay in naming the color of this word, if color and color-word do not match. This test is used for registration of the color-word interference tendency, i.e. impairment of the reading speed or color recognition due to interfering information. Therefore, it is useful in determining the individual susceptibility to stimulus disturbing mental processes		0.00 (-0.40,0.40)
						Higher-order functions	The Comparing of Names Test	It consists of two columns of words (names). The respondent decides whether couples of words (names) in both columns are exactly the same. This test is designed to measure the ability to see pertinent detail in verbal material		-0.04 (-0.44,0.36)

### Risk of bias and quality of evidence assessment

**Fig. 2:**
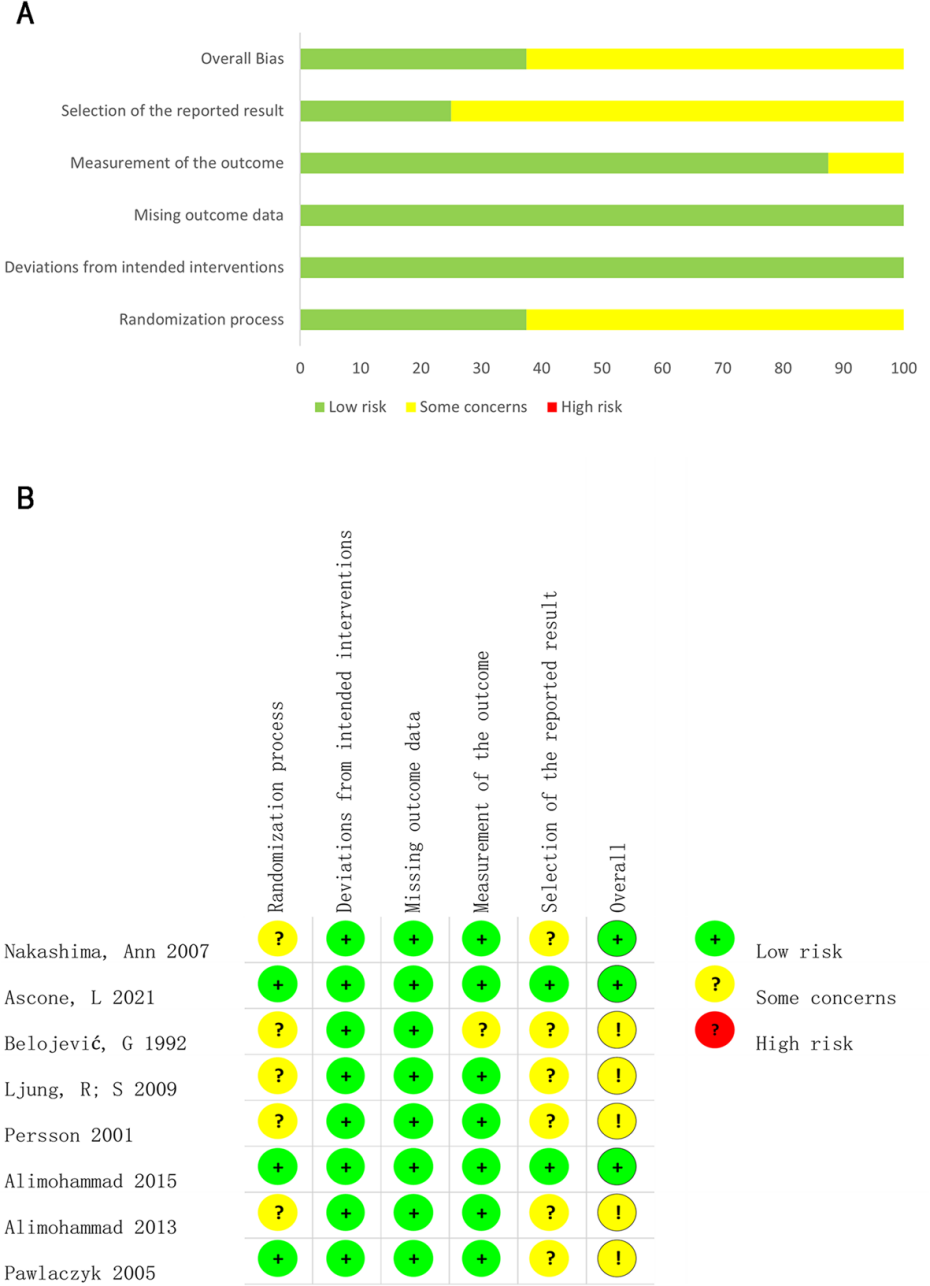
Risk of Bias of individual studies

Risk of bias assessment conducted by two authors revealed a moderate risk of overall bias in the included studies quality. In terms of overall evaluation, three studies were considered to have a low risk of bias, whereas five were considered to have an uncertain risk of bias. Major uncertainties were noted in the methods of random assignment and selective reporting of study results. The risk of bias in different domains, both in terms of percentages and summary, is shown in Fig. 2. Detailed assessment data on the risk of bias can be found in Additional file 5. The GRADE system provided information about the certainty of the conclusions and strength of evidence. All estimates were of low or very low quality and the decisions made and reasons for these decisions are specified in Additional file 6.

### Impact of LFN exposure on cognitive function

From the perspective of cognitive psychology, cognitive function can be divided into basic and higher-order cognitive functions. Basic cognitive functions include attention, execution, and memory, while higher-order cognitive functions include textual reasoning, decision-making ability, and innovation [39, 40]. Compared to basic cognitive function, in the process of higher-order cognitive function, more brain regions utilize more resources, with the prefrontal cortex and cerebellum playing a core role [41, 42]. For analysis, we subdivided cognitive functions into four domains as mentioned above. The results for each domain are described as follows:

### Impact of LFN exposure on attentional functioning

**Fig. 3:**
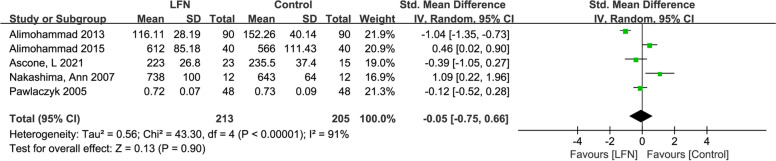
Forest plot of the effect of LFN on Attentional functioning domain

Among the eight included studies, five involved the cognitive function domain of attention. Figure 3 shows high heterogeneity among the studies (*p* < 0.001, I^2^ = 91%, Tau^2^ = 0.56). A random model was utilized for the meta-analysis, revealing no statistically significant impact of LFN intervention on changes in attention levels (Z = 0.13, *p* = 0.90), with the SMD being -0.05 (95% CI: -0.75, 0.66).

### Impact of LFN exposure on executive functioning

**Fig. 4:**
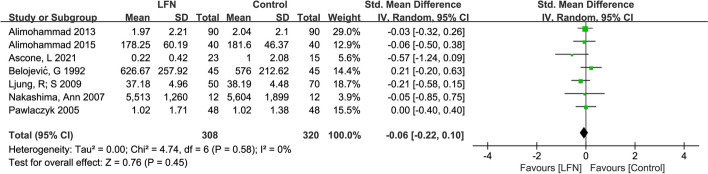
Forest plot of the effect of LFN on Executive functioning domain

Seven of the eight included studies evaluated executive functioning and were analyzed as shown in Fig. 4. The studies exhibited no heterogeneity (*p* = 0.58, I^2^ = 0%, Tau^2^ = 0.00). A random-effects model was employed for the meta-analysis, and results revealed that the impact of LFN intervention on changes in attention levels was not statistically significant (Z = 0.76, *p* = 0.45), with an SMD of -0.06 (95%CI: -0.22, 0.10).

### Impact of LFN exposure on memory

**Fig. 5:**
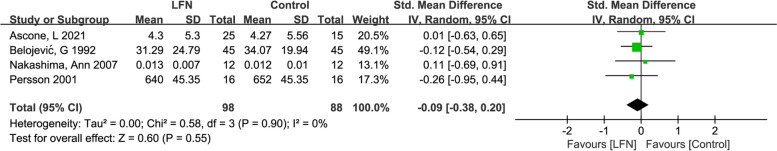
Forest plot of the effect of LFN on Memory domain

Four of the eight included studies were related to memory and analyzed as shown in Fig. 5. The studies demonstrated no heterogeneity (*p* = 0.90, I^2^ = 0%, Tau^2^ = 0.00). A random-effects model was employed for meta-analysis, revealing that the impact of LFN intervention on changes in attention levels was not statistically significant (Z = 0.60, *p* = 0.55) and demonstrating an SMD of -0.09 (95% CI: -0.38, 0.20).

### Impact of LFN exposure on higher-order functions

**Fig. 6:**
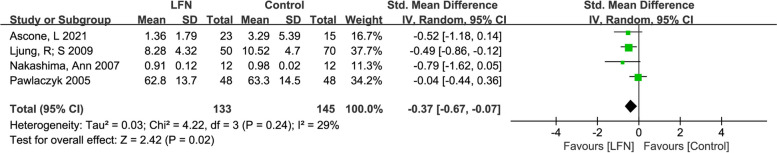
Forest plot of the effect of LFN on Higher-order functions domain

Four of the eight included studies discussed higher-order functions and were subsequently analyzed, as shown in Fig. 6. The studies demonstrated moderate heterogeneity (*p* = 0.24, I^2^ = 29%, Tau^2^ = 0.03). A random-effects model was utilized for the meta-analysis, indicating that LFN intervention had a negative impact on higher-order cognitive functions, with this difference being statistically significant (Z = 2.42, *p* = 0.02), and an SMD of -0.37 (95% CI: -0.67, -0.07).

## Discussion

### Summary of evidence

Noise is a serious global public health problem that deserves our attention for its impact on human life and health. Our initial literature search revealed numerous studies on noise and the relationship between noise and cognitive functions. Nevertheless, most of these studies primarily focused on environmental noise, traffic noise, or medium- to high- frequency noise in a broader sense [43–45]. The volume of studies specifically addressing LFN was notably limited, and views on the impact of LFN on cognitive functions were diverse. Some studies have suggested that LFN exposure negatively affects cognitive functions [46–49], whereas others have suggested no such effect [13]. Some even proposed that LFN exposure can enhance cognitive function [50]. To deepen our understanding of the relationship between LFN and cognitive function, we conducted a meta-analysis.

Cognitive function is a broad and complex concept, therefore, assessing changes in cognitive function from an overall perspective is likely to be one-sided and subjective and could lead to significant heterogeneity issues. To better assess the relationship between LFN exposure and cognitive function, we divided cognitive function into four domains mentioned earlier [39]. In the present study, we evaluated a total of eight studies: five on attention, seven on executive functioning, four on memory, and four on higher-order cognitive functions.

Our meta-analysis concluded that there was a moderate risk of bias and low quality of evidence indicating a negative impact of LFN exposure on higher-order cognitive functions (such as logical reasoning, mathematical calculation, and data processing), with low heterogeneity among the results of related studies. This suggests that responses to LFN within the domain of higher-order cognitive functions are relatively consistent. Previous studies have demonstrated that low-frequency traffic noise slows down reading speed and negatively impacts performance in mathematical tasks [48] and that LFN can reduce mental arithmetic test accuracy [47]. Gao et al. [51] observed a significant longitudinal negative correlation between traffic noise exposure and mathematical performance in cognitive testing. We have demonstrated in a previous study that participants exposed to LFN experience higher levels of cognitive load [52]. Further, LFN exposure does not significantly change basic cognitive functions, such as attention, executive function, and memory. There was no heterogeneity between the results of relevant studies in the domains of executive function and memory, but heterogeneity was higher within the attention domain, which aligns with the findings of Thompson et al. [52], who demonstrated that noise exposure worsens cognitive impairment and reading ability but has no effect on executive function. This may be because basic cognitive functions are more readily achieved and that the influence of LFN is not substantial enough to affect these simpler cognitive functions. Alternatively, the impact of LFN on basic cognitive functions could be too small for our selected detection indicators to determine [39]. We noted that the impact of LFN on cognitive functions may also be related to each individual’s noise sensitivity, which could potentially be the primary reason for changes in cognitive function [6, 26, 53].

### Limitations

In this study, we summarized literature concerning the relationship between LFN and cognition in the general (non-pathological) population up until December 2022. Two authors independently used rigorous scientific methods to assess the risk of bias. We categorized cognitive function into four domains and analyzed each, specifically focusing on LFN, an issue of increasing public health interest. Our findings revealed that, while LFN interventions may not substantially impact basic cognitive function, they could potentially negatively affect higher-order cognitive functions. These results are useful in the development of protective measures against LFN.

This study has some limitations. First, because research solely addressing LFN is relatively scarce and the cognitive tasks among studies are different, standardizing the exposure dose of LFN, including frequency, sound pressure level, exposure method, and intervention time, when incorporating the literature is challenging. This could have potentially affected the quality of evidence concerning the relationship between LFN and cognitive function. Second, cognitive function is broad, and representing changes in cognitive function from a single perspective may be somewhat one-sided. Even though we categorized and analyzed cognitive function, the summarization process may still be subjective. Finally, because of limitations in LFN research, the number of studies and population covered in our study were limited. Meanwhile, considering the GRADE assessment of current evidence is of poor quality, the conclusions demonstrated herein cannot be generalized.

### Future research directions

The impact of LFN on basic cognitive functions may be too small to detect or may depend on individual noise sensitivity. Studies delving into the impact of LFN on cognitive function are lacking, with the scope of populations involved in these studies being relatively narrow. First, to conduct research of higher quality on the relationship between LFN interventions and cognitive functions, it is of paramount importance to conduct multi-center studies that comprise the entire population and include larger sample sizes.

Second, a unified standard for the methods and intensity of LFN interventions is yet to be established, and the selection of methods to evaluate cognitive function also varies. Although our study carefully selected situations involving LFN interventions and classified domains of cognitive function, the presence of confounding factors still poses a limitation on comparability across studies.

Third, given the potential role of noise sensitivity in its impact on cognitive function, future research demands a more profound exploration of this contributing factor. Furthermore, it should be incorporated into the inclusion criteria for study participants in a standardized manner.

Finally, it is hoped that the protection standards for LFN can be improved, and the subjective protection awareness of long-term exposure groups to actively protect against LFN can be improved, such as wearing noise reducing earphones and controlling the duration of noise exposure.

## Conclusions

Through this meta-analysis, we aimed to explore the influence of LFN intervention on cognitive function. Our research indicated that, to date, there is no evidence supporting the notion that LFN intervention impacts attention levels, executive function, and memory. However, there is evidence of low quality showing that LFN intervention may lead to a reduction in higher-order cognitive functions, including reasoning and mathematical calculation. This impact may be associated with the level of cognitive load and susceptibility to noise. When the future research is conducted, these two factors and their interventions must be considered. In practical scenarios, attention should be directed toward the negative consequences of LFN during noise protection, and relevant protective measures should be implemented.

### Supplementary Information

**Additional file 1. **The PRISMA 2020 checklist of this review.

**Additional file 2. **The Literature Search Strategy of this review.

**Additional file 3. **Detailed information for effect sizes transformation.

**Additional file 4. **Exclusion reasons for 42 full-text articles.

**Additional file 5. **The individual study bias analyses of this review.

**Additional file 6. **GRADE summary for quality of evidence from LFN associated with cognitive function.

## Data Availability

The datasets used and/or analysed during the current study are available from the corresponding author on reasonable request.

## Abbreviations

LFN: Low-frequency noise
SMD: Standardized mean difference
CI: Confidence interval
PECOS: Participants, exposure, control/comparison, outcomes, and study design
RoB 2: Revised Cochrane risk of bias tool for randomized trials
GRADE: Grading of Recommendations Assessment, Development, and Evaluation
