# Supplementary material for: Effect of low-frequency noise exposure on cognitive function: a systematic review and meta-analysis
Source: BMC Public Health. 2024 Jan 9;24:125. doi: 10.1186/s12889-023-17593-5 (PMC10775542; doi:10.1186/s12889-023-17593-5)
Supplement: Supplementary file 2 — Additional file 2. The Literature Search Strategy of this review. [file 12889_2023_17593_MOESM2_ESM.docx]

**Additional file 2. The Literature Search Strategy of this review.**

| **Table S2.1 Literature Search Strategy for Database PubMed (Through Dec 16, 2022)** | | |
| --- | --- | --- |
|  | **Search Terms** | **Results** |
| #1 | low frequency noise[Title/Abstract] OR "low frequency sound"[Title/Abstract] OR "infrasound"[Title/Abstract] | 1465 |
| #2 | noise[Title/Abstract] OR "sound"[Title/Abstract] | 246718 |
| #3 | "air-conditioning"[Title/Abstract] OR (("variabilities"[All Fields] OR "variability"[All Fields] OR "variable"[All Fields] OR "variable s"[All Fields] OR "variables"[All Fields] OR "variably"[All Fields]) AND "distribution equipment"[Title/Abstract]) OR "elevator"[Title/Abstract] OR "ventilator"[Title/Abstract] OR "ventilation"[Title/Abstract] OR "wind turbine"[Title/Abstract] OR "wind farm"[Title/Abstract] OR "transformer substation"[Title/Abstract] OR (("convertor"[All Fields] OR "convertors"[All Fields]) AND "station"[Title/Abstract]) OR "infrastructure"[Title/Abstract] OR "compressor"[Title/Abstract] OR "power lines"[Title/Abstract] OR "transmission lines"[Title/Abstract] OR "electrical installation"[Title/Abstract] OR "refrigerator"[Title/Abstract] OR "cooling tower"[Title/Abstract] OR "sewerage"[Title/Abstract] OR "residential"[Title/Abstract] OR "domestic"[Title/Abstract] OR "underground garage"[Title/Abstract] OR "engine"[Title/Abstract] OR "construction"[Title/Abstract] OR "traffic"[Title/Abstract] | 558178 |
| #4 | #2 AND #3 | 7683 |
| #5 | "low frequency noise"[Title/Abstract] OR "low frequency sound"[Title/Abstract] OR "infrasound"[Title/Abstract] OR (("Noise"[Title/Abstract] OR "sound"[Title/Abstract]) AND ("air-conditioning"[Title/Abstract] OR (("variabilities"[All Fields] OR "variability"[All Fields] OR "variable"[All Fields] OR "variable s"[All Fields] OR "variables"[All Fields] OR "variably"[All Fields]) AND "distribution equipment"[Title/Abstract]) OR "elevator"[Title/Abstract] OR "ventilator"[Title/Abstract] OR "ventilation"[Title/Abstract] OR "wind turbine"[Title/Abstract] OR "wind farm"[Title/Abstract] OR "transformer substation"[Title/Abstract] OR (("convertor"[All Fields] OR "convertors"[All Fields]) AND "station"[Title/Abstract]) OR "infrastructure"[Title/Abstract] OR "compressor"[Title/Abstract] OR "power lines"[Title/Abstract] OR "transmission lines"[Title/Abstract] OR "electrical installation"[Title/Abstract] OR "refrigerator"[Title/Abstract] OR "cooling tower"[Title/Abstract] OR "sewerage"[Title/Abstract] OR "residential"[Title/Abstract] OR "domestic"[Title/Abstract] OR "underground garage"[Title/Abstract] OR "engine"[Title/Abstract] OR "construction"[Title/Abstract] OR "traffic"[Title/Abstract])) | 9032 |
| #6 | #1 OR #4 | 2733146 |
| #7 | #5 AND #6 | 1702 |
| #8 | Limit #7 to English Language | 1640 |

| **Table S2.2 Literature Search Strategy for Database Medline (Through Dec 16, 2022)** | | |
| --- | --- | --- |
|  | **Search Terms** | **Results** |
| #1 | (Low frequency noise or low frequency sound or infrasound).ab,ti. | 1619 |
| #2 | (noise or sound).ab,ti. | 299282 |
| #3 | (air-conditioning or variable distribution equipment or elevator or ventilator or ventilation or wind turbine or wind farm or transformer substation or convertor station or infrastructure or compressor or power lines or transmission lines or electrical installation or refrigerator or cooling tower or sewerage or residential or domestic or underground garage or engine or construction or traffic).ab,ti. | 711473 |
| #4 | #2 AND #3 | 9204 |
| #5 | (cognition or cognitive function or cognitive impairment or cognitive or neurodevelopment or memory or attention or attentional functioning or executive function or neuropsychology or neuropsychological or neurobehavior or neurobehavioral or intelligence or IQ or psychomotor or verbal or visual spatial or visual motor or academic or learning or reading or reaction time or accuracy or reasoning or flexibility or problem-solving or processing speed or mental workload or mental).ab,ti. | 3902596 |
| #6 | #1 OR #4 | 10699 |
| #7 | #5 AND #6 | 2165 |
| #8 | Limit #7 to English Language | 2103 |
| #9 | Limit #8 to Humans | 1326 |

| **Table S2.3 Literature Search Strategy for Database Web of Science (Through Dec 16, 2022)** | | |
| --- | --- | --- |
|  | **Search Terms** | **Results** |
| #1 | TS=(Low frequency noise or low frequency sound or infrasound) | 45410 |
| #2 | TS=(noise or sound) | 515594 |
| #3 | TS=(air-conditioning or variable distribution equipment or elevator or ventilator or ventilation or wind turbine or wind farm or transformer substation or convertor station or infrastructure or compressor or power lines or transmission lines or electrical installation or refrigerator or cooling tower or sewerage or residential or domestic or underground garage or engine or construction or traffic) | 1335761 |
| #4 | #2 AND #3 | 36290 |
| #5 | TS=(cognition or cognitive function or cognitive impairment or cognitive or neurodevelopment or memory or attention or attentional functioning or executive function or neuropsychology or neuropsychological or neurobehavior or neurobehavioral or intelligence or IQ or psychomotor or verbal or visual spatial or visual motor or academic or learning or reading or reaction time or accuracy or reasoning or flexibility or problem-solving or processing speed or mental workload or mental) | 4309146 |
| #6 | #1 OR #4 | 78488 |
| #7 | #5 AND #6 | 16431 |
| #8 | Limit #7 to Humans | 2459 |

| **Table S2.4 Literature Search Strategy for Database PsychInfo (Through Dec 16, 2022)** | | |
| --- | --- | --- |
|  | **Search Terms** | **Results** |
| #1 | (abstract(Low frequency noise OR low frequency sound OR infrasound) OR (abstract(noise OR sound) AND abstract(air-conditioning OR variable distribution equipment OR elevator OR ventilator OR ventilation OR wind turbine OR wind farm OR transformer substation OR convertor station OR infrastructure OR compressor OR power lines OR transmission lines OR electrical installation OR refrigerator OR cooling tower OR sewerage OR residential OR domestic OR underground garage OR engine OR construction OR traffic))) AND abstract(cognition OR cognitive function OR cognitive impairment OR cognitive OR neurodevelopment OR memory OR attention OR attentional functioning OR executive function OR neuropsychology OR neuropsychological OR neurobehavior OR neurobehavioral OR intelligence OR IQ OR psychomotor OR verbal OR visual spatial OR visual motor OR academic OR learning OR reading OR reaction time OR accuracy OR reasoning OR flexibility OR problem-solving OR processing speed OR mental workload OR mental).ab | 6391 |
| #2 | Limit #1 to Scholarly Journals (Source Type) | 4432 |
| #3 | Limit #2 to Humans | 646 |
| #4 | Limit #3 to Article (Document Type) | 644 |
| #5 | Limit #4 to English Language | 638 |
