# Supplementary material for: Effect of low-frequency noise exposure on cognitive function: a systematic review and meta-analysis
Source: BMC Public Health. 2024 Jan 9;24:125. doi: 10.1186/s12889-023-17593-5 (PMC10775542; doi:10.1186/s12889-023-17593-5)
Supplement: Supplementary file 3 — Additional file 3. Detailed information for effect sizes transformation. [file 12889_2023_17593_MOESM3_ESM.docx]

**Additional file 3. Detailed information for effect sizes transformation.**

| Studies | Original dates (LFN_mean±SD /Control_mean±SD) | Dates source location | | Transformation | Effect sizes in meta-analysis SMD (95%CI) |
| --- | --- | --- | --- | --- | --- |
| Nakashima, Ann 2007 | Attentional functioning:738±100/643±64  (Serial reaction time task)  Executive unctioning:5513±1260/5604±1899  (Mental addition task)  Memory:0.013±0.007/0.012±0.01  (Detection of repeated numbers task)  Higher-order functions:0.91±0.12/0.98±0.02  (Logical reasoning task) | | Table 8,Table 3,Table 7 and Table 2 in this paper. | $d(SMD)=\frac{\bar{x_{1}}-\bar{x}_{2}}{s_{c}}\left( 1-\frac{3}{4N-9} \right)$*;* **^#1^**  $s_{c}=\sqrt{\frac{s_{1}^{2}\left( n_{1}-1 \right)+s_{2}^{2}\left( n_{2}-1 \right)}{\left( n_{1}+n_{2}-2 \right)}}$ **^#2^** | Attentional functioning:  1.09 (0.22,1.96)  Executive functioning:  -0.05 (-0.85,0.75)  Memory:  0.11 (-0.69,0.91)  Higher-order functions:  -0.79 (-1.62,0.05) |
| Ascone, L 2021 | Attentional functioning:223±26.8/235.5±37.4  (Alertness task)  Executive functioning:0.22±0.42/1.0±2.08  (GoNoGo task)  Memory:4.3± 5.3/4.27±5.56  (Sustaine attention task)  Higher-order functions:1.36±1.79/3.29 ±5.39  (Covert Shift of Attention task) | | Appendix III – Descriptive pre-post data (means and standard deviations) for all behavioral variables. | $d(SMD)=\frac{\bar{x_{1}}-\bar{x}_{2}}{s_{c}}\left( 1-\frac{3}{4N-9} \right)$*;* **^#1^**  $s_{c}=\sqrt{\frac{s_{1}^{2}\left( n_{1}-1 \right)+s_{2}^{2}\left( n_{2}-1 \right)}{\left( n_{1}+n_{2}-2 \right)}}$ **^#2^** | Attentional functioning:  -0.39 (1.05,0.27)  Executive functioning:  -0.57 (-1.24,0.09)  Memory:  0.01 (-0.63,0.65)  Higher-order functions:  -0.52 (-1.18,0.14) |
| Belojević, G 1992 | Executive functioning:  Tolerant_587±275/527±144;  Neutral_601±218/598± 262;  Sensitive_692±281/603±221  (Mental arithmetic task)  Memory:  Tolerant_24.7±27.1/25.5±17.4;  Neutral_27.3±23.1/32.7±17.9;  Sensitive_44.0±20.6/44.0±21.0  (Short-term memory task) | | Table 2 and Table 5 in this paper. According to the formula, we merged subgroups. | $SD=\sqrt{\frac{\left( N_{1}-1 \right)SD_{1}^{2}+\left( N_{2}-1 \right)SD_{2}^{2}+\frac{N_{1}N_{2}}{N_{1}+N_{2}}\left( M_{1}^{2}+M_{2}^{2}-2M_{1}M_{2} \right)}{N_{1}+N_{2}-1}}$**^#3^** | Executive functioning:  0.21 (-0.20,0.63)  Memory:  -0.12 (-0.54,0.29) |
| Ljung, R 2009 | Executive functioning:37.18±4.96/38.19±4.48  (Reading test task)  Higher-order functions:8.28±4.32/10.52 ±4.7  (Basic mathematics task) | | According to the results section of the article. |  | Executive functioning:  -0.21 (-0.58,0.15)  Higher-order functions:  -0.49 (-0.86, -0.12) |
| Persson 2001 | Memory:  mean_640/652,  p-value_0.46  (Short-time memory task) | | Missing data were obtained by contacting the corresponding authors through email. And convert the standard deviation based on the mean and p-value through the formula. | $SD=\frac{SE}{\sqrt{1/NE+1/NC}} ; SE=MD/t$ **^#4^**  $d(SMD)=\frac{\bar{x_{1}}-\bar{x}_{2}}{s_{c}}\left( 1-\frac{3}{4N-9} \right)$*;* **^#1^**  $s_{c}=\sqrt{\frac{s_{1}^{2}\left( n_{1}-1 \right)+s_{2}^{2}\left( n_{2}-1 \right)}{\left( n_{1}+n_{2}-2 \right)}}$ **^#2^** | Memory:  -0.26 (-0.95,0.44) |
| Alimohammad 2015 | Attentional functioning:  Introvert_617±81/590±104;  Extrovert_607±91/542± 116  (Reaction time)  Executive functioning:  Introvert_206.3±68/203.7±53;  Extrovert_150.2±34/159.5± 24  (Movement time) | | According to dates from the results section in the paper and the special formula，we merged subgroups. | $SD=\sqrt{\frac{\left( N_{1}-1 \right)SD_{1}^{2}+\left( N_{2}-1 \right)SD_{2}^{2}+\frac{N_{1}N_{2}}{N_{1}+N_{2}}\left( M_{1}^{2}+M_{2}^{2}-2M_{1}M_{2} \right)}{N_{1}+N_{2}-1}}$**^#3^** | Attentional functioning:  0.46 (0.02,0.90)  Executive functioning:  -0.06 (-0.50,0.38) |
| Alimohammad 2013 | Attentional functioning:116.11±28.19/152.26±40.14  (Cognitrone task)  Executive functioning:1.97±2.21/2.04±2.1  (Stroop task) | | According to the results section of the article (table 1). | $d(SMD)=\frac{\bar{x_{1}}-\bar{x}_{2}}{s_{c}}\left( 1-\frac{3}{4N-9} \right)$*;* **^#1^**  $s_{c}=\sqrt{\frac{s_{1}^{2}\left( n_{1}-1 \right)+s_{2}^{2}\left( n_{2}-1 \right)}{\left( n_{1}+n_{2}-2 \right)}}$ **^#2^** | Attentional functioning:  -0.14 (-1.35,-0.73)  Executive functioning:  -0.03 (-0.32,0.26) |
| Pawlaczyk 2005 | Attentional functioning:0.72±0.07/0.73±0.09  (Continuous Attention Test)  Executive functioning:1.02±1.71/1.02±1.38  (Stroop Color-Word Test)  Higher-order functions:62.8±13.7/63.3 ±14.5  (Comparing of Names Test) | | According to the tables in the results section of the article. |  | Attentional functioning:  -0.12 (-0.52,0.28)  Executive functioning:  -0.00 (-0.40,0.40)  Higher-order functions:  -0.04 (-0.67, -0.07) |

**^#1 #2^** SMD: Standardized mean difference; N/n: number of samples;‾x：mean; S: standard deviation.

**^#3^** SD: Standard deviation.; N: number of samples.

**^#4^** SD: Standard deviation.; SE: standard error; NE: number of experimental group samples; NC: number of control group samples; MD: mean difference between groups;

Ref: Luo J, Leng W. Theory & practice of systematic review/meta-analysis[M]. Military Medical Science Press, 2013
