# Supplementary material for: Effect of low-frequency noise exposure on cognitive function: a systematic review and meta-analysis
Source: BMC Public Health. 2024 Jan 9;24:125. doi: 10.1186/s12889-023-17593-5 (PMC10775542; doi:10.1186/s12889-023-17593-5)
Supplement: Supplementary file 4 — Additional file 4. Exclusion reasons for 42 full-text articles. [file 12889_2023_17593_MOESM4_ESM.docx]

**Additional file 4.** **Exclusion reasons for 42 full-text articles.**

| Author | Year | Title | Exclued Reasons |
| --- | --- | --- | --- |
| Kaczmarska et al | 2007 | A study of annoyance caused by low-frequency noise during mental work. | Does not measure cognition as a single concept |
| Gong et al | 2022 | Association between Noise Annoyance and Mental Health Outcomes: A Systematic Review and Meta-Analysis | Review article |
| Weichenberger et al | 2015 | Brief bursts of infrasound may improve cognitive function--an fMRI study | Type of study not relevant or study not LFN/cognition-related |
| Jalali et al | 2016 | Changes in quality of life and perceptions of general health before and after operation of wind turbines | Does not measure cognition as a single concept |
| Jafar et al | 2018 | Chronic traffic noise stress accelerates brain impairment and cognitive decline in mice. | Animal article |
| Schäffer et al | 2022 | Does the Macro-Temporal Pattern of Road Traffic Noise Affect Noise Annoyance and Cognitive Performance? | Does not measure LFN as a single concept |
| Nissenbaum et al | 2012 | Effects of industrial wind turbine noise on sleep and health | Does not measure cognition as a single concept |
| Yuan et al | 2009 | Effects of infrasound on hippocampus-dependent learning and memory in rats and some underlying mechanisms | Animal article |
| Elmenhorst et al | 2010 | Effects of nocturnal aircraft noise on cognitive performance in the following morning: dose-response relationships in laboratory and field | Does not measure LFN as a single concept |
| Fan et al | 2022 | Effects of Noise Exposure and Mental Workload on Physiological Responses during Task Execution | Does not measure LFN as a single concept |
| Key et al | 1981 | Effects of noise frequency on performance and annoyance for women and men | Does not measure cognition as a single concept |
| Song et al | 2022 | Effects of Noise Type and Noise Sensitivity on Working Memory and Noise Annoyance. | Does not measure LFN as a single concept |
| Pawlaczyk-Luszczynska et al | 2010 | Evaluation of annoyance from low frequency noise under laboratory conditions | Does not measure LFN as a single concept |
| Pawlaczyk-Łuszczyńska et al | 2014 | Evaluation of annoyance from the wind turbine noise: a pilot study | Does not measure LFN as a single concept |
| Schapkin et al | 2006 | Executive brain functions after exposure to nocturnal traffic noise: effects of task difficulty and sleep quality | Does not measure LFN as a single concept |
| Ohlenforst et al | 2016 | Exploring the Relationship Between Working Memory, Compressor Speed, and Background Noise Characteristics | Does not measure LFN as a single concept |
| Liu et al | 2022 | Extensive hearing loss induced by low-frequency noise exposure | Does not measure cognition as a single concept |
| Baliatsas et al | 2016 | Health effects from low-frequency noise and infrasound in the general population: Is it time to listen? A systematic review of observational studies | Review article |
| Schmidt et al | 2014 | Health effects related to wind turbine noise exposure: a systematic review | Review article |
| van Kamp et al | 2021 | Health Effects Related to Wind Turbine Sound: An Update | Does not measure cognition as a single concept |
| Sandrock et al | 2009 | Impairing effects of noise in high and low noise sensitive persons working on different mental tasks | Does not measure LFN as a single concept |
| Jeffery et al | 2014 | Industrial wind turbines and adverse health effects | Review article |
| Umemura et al | 1992 | Influence of noise on heart rate and quantity of work in mental work | Does not measure LFN as a single concept |
| Di et al | 2018 | Influences of combined traffic noise on the ability of learning and memory in mice | Animal article |
| Weuve et al | 2021 | Long-term community noise exposure in relation to dementia, cognition, and cognitive decline in older adults | Does not measure LFN as a single concept |
| Sandrock et al | 2010 | Mental strain and annoyance during cognitive performance in different traffic noise conditions | Does not measure LFN as a single concept |
| Lenc et al | 2018 | Neural tracking of the musical beat is enhanced by low-frequency sounds | Does not measure LFN as a single concept |
| Braat-Eggen et al | 2017 | Noise disturbance in open-plan study environments: a field study on noise sources, student tasks and room acoustic parameters | Does not measure LFN as a single concept |
| Ziaran et al | 2013 | Potential health effects of standing waves generated by low frequency noise | Does not measure cognition as a single concept |
| Lee et al | 2013 | Relating traffic, construction, and ventilation noise to cognitive performances and subjective perceptions | Does not measure LFN as a single concept |
| Harris et al | 1976 | Review of the effects of infrasound on man | Review article |
| Mac et al | 2021 | Road traffic noise and cognitive function in older adults: a cross-sectional investigation of The Irish Longitudinal Study on Ageing | Does not measure LFN as a single concept |
| Fuks et al | 2019 | Road Traffic Noise at the Residence, Annoyance, and Cognitive Function in Elderly Women | Does not measure LFN as a single concept |
| Tangermann et al | 2022 | The association of road traffic noise with cognition in adolescents: A cohort study in Switzerland | Does not measure LFN as a single concept |
| Javadi et al | 2022 | The Effect of Low Frequency Noises Exposure on the Precision of Human at the Mathematical Tasks | No useful data |
| Anund et al | 2015 | The effect of low-frequency road noise on driver sleepiness and performance. | Does not measure cognition as a single concept |
| Khajehnasiri et al | 2022 | The effect of traffic noise on cognitive performance with regard to personality traits: A laboratory experiment study | Does not measure LFN as a single concept |
| Soderlund et al | 2010 | The effects of background white noise on memory performance in inattentive school children | Does not measure LFN as a single concept |
| Shield et al | 2008 | The effects of environmental and classroom noise on the academic attainments of primary school children | Does not measure LFN as a single concept |
| Alimohammad et al | 2013 | The effects of road traffic noise on mental performance | Does not measure LFN as a single concept |
| Schlittmeier et al | 2015 | The impact of road traffic noise on cognitive performance in attention-based tasks depends on noise level even within moderate-level ranges | Does not measure LFN as a single concept |
| Remacle et al | 2012 | Vocal impact of a prolonged reading task at two intensity levels: Objective measurements and subjective self-ratings | Type of study not relevant or study not LFN/cognition-related |
