# Supplementary material for: Effect of low-frequency noise exposure on cognitive function: a systematic review and meta-analysis
Source: BMC Public Health. 2024 Jan 9;24:125. doi: 10.1186/s12889-023-17593-5 (PMC10775542; doi:10.1186/s12889-023-17593-5)
Supplement: Supplementary file 5 — Additional file 5. The individual study bias analyses of this review. [file 12889_2023_17593_MOESM5_ESM.docx]

**Additional file 5. The individual study bias analyses of this review.**

| **Nakashima A, Abel SM, Duncan M, Smith D. Hearing, communication and cognition in low-frequency noise from armoured vehicles. Noise Health. 2007 Apr-Jun;9(35):35-41. doi: 10.4103/1463-1741.36978. PMID: 18025753. (Study 1)** | | | | | | | | | | |  |  |
| --- | --- | --- | --- | --- | --- | --- | --- | --- | --- | --- | --- | --- |
| **Domain** | | **Signalling question** | | | **Response** | | | | | **Comments** |  |  |
| **Bias arising from the randomization process** | | 1.1 Was the allocation sequence random? | | | NI | | | | | The only information about randomization methods is a statement that the study is randomized. |  |  |
|  |  | 1.2 Was the allocation sequence concealed until participants were enrolled and assigned to interventions? | | | NI | | | | |  |  |  |
|  |  | 1.3 Did baseline differences between intervention groups suggest a problem with the randomization process? | | | N | | | | | No imbalances are apparent or if any observed imbalances are compatible with chance. |  |  |
|  |  | **Risk of bias judgement** | | | **Some concerns** | | | | | RoB 2 generates itself based on each of the above judgments. |  |  |
| **Bias due to deviations from intended interventions** | | 2.1.Were participants aware of their assigned intervention during the trial? | | | PY | | | | | Participants experienced some effects that they knew to be exposed to LFN. |  |  |
|  |  | 2.2.Were carers and people delivering the interventions aware of participants' assigned intervention during the trial? | | | Y | | | | | Carers or people delivering the interventions knew to be specific to one of the interventions. |  |  |
|  |  | 2.3. If Y/PY/NI to 2.1 or 2.2: Were there deviations from the intended intervention that arose because of the experimental context? | | | N | | | | | Changes to intervention are consistent with the trial protocol |  |  |
|  |  | 2.4 If Y/PY to 2.3: Were these deviations likely to have affected the outcome? | | | NA | | | | | Not applicable |  |  |
|  |  | 2.5. If Y/PY/NI to 2.4: Were these deviations from intended intervention balanced between groups? | | | NA | | | | | Not applicable |  |  |
|  |  | 2.6 Was an appropriate analysis used to estimate the effect of assignment to intervention? | | | Y | | | | | Postrandomization exclusions of ineligible participants can be considered appropriate. |  |  |
|  |  | 2.7 If N/PN/NI to 2.6: Was there potential for a substantial impact (on the result) of the failure to analyse participants in the group to which they were randomized? | | | NA | | | | | Not applicable |  |  |
|  |  | **Risk of bias judgement** | | | **Low** | | | | | RoB 2 generates itself based on each of the above judgments. |  |  |
| **Bias due to missing outcome data** | | 3.1 Were data for this outcome available for all, or nearly all, participants randomized? | | | PY | | | | | The appropriate study population for an analysis of the effect of LFN is all randomized participants. |  |  |
|  |  | 3.2 If N/PN/NI to 3.1: Is there evidence that result was not biased by missing outcome data? | | | NA | | | | | Not applicable |  |  |
|  |  | 3.3 If N/PN to 3.2: Could missingness in the outcome depend on its true value? | | | NA | | | | | Not applicable |  |  |
|  |  | 3.4 If Y/PY/NI to 3.3: Is it likely that missingness in the outcome depended on its true value? | | | NA | | | | | Not applicable |  |  |
|  |  | **Risk of bias judgement** | | | **Low** | | | | | RoB 2 generates itself based on each of the above judgments. |  |  |
| **Bias in measurement of the outcome** | | 4.1 Was the method of measuring the outcome inappropriate? | | | N | | | | | For pre-specified outcomes, the answer to this question will be ‘No’ or ‘Probably no. |  |  |
|  |  | 4.2 Could measurement or ascertainment of the outcome have differed between intervention groups? | | | N | | | | | Having used the same measurement. |  |  |
|  |  | 4.3 Were outcome assessors aware of the intervention received by study participants? | | | PN | | | | | Outcome assessors were blinded to intervention status. |  |  |
|  |  | 4.4 If Y/PY/NI to 4.3: Could assessment of the outcome have been influenced by knowledge of intervention received? | | | NA | | | | | Not applicable |  |  |
|  |  | 4.5 If Y/PY/NI to 4.4: Is it likely that assessment of the outcome was influenced by knowledge of intervention received? | | | NA | | | | | Not applicable |  |  |
|  |  | **Risk of bias judgement** | | | **Low** | | | | | RoB 2 generates itself based on each of the above judgments. |  |  |
| **Bias in selection of the reported result** | | 5.1 Were the data that produced this result analysed in accordance with a pre-specified analysis plan that was finalized before unblinded outcome data were available for analysis? | | | NI | | | | | The researchers’ pre-specified intentions are not available in detail. |  |  |
|  |  | 5.2 ... multiple eligible outcome measurements (e.g. scales, definitions, time points) within the outcome domain? | | | PN | | | | | There is clear evidence (usually through examination of a trial protocol or statistical analysis plan) that all eligible reported results for the outcome domain correspond to all intended outcome measurements. |  |  |
|  |  | 5.3 ... multiple eligible analyses of the data? | | | N | | | | |  |  |  |
|  |  | **Risk of bias judgement** | | | **Some concerns** | | | | | RoB 2 generates itself based on each of the above judgments. |  |  |
| **Overall bias** | | **Risk of bias judgement** | | | **Low** | | | | | RoB 2 generates itself based on the judgment of each domain above. |  |  |
|  | |  | |  | | |  | |  |  |  |  |
|  | |  | |  | | |  | |  |  |  |  |
| **Ascone L, Kling C, Wieczorek J, Koch C, Kühn S. A longitudinal, randomized experimental pilot study to investigate the effects of airborne infrasound on human mental health, cognition, and brain structure. Sci Rep. 2021 Feb 4;11(1):3190. doi: 10.1038/s41598-021-82203-6. PMID: 33542290; PMCID: PMC7862356. (Study 2)** | | | | | | | | | | |  |  |
| **Domain** | | **Signalling question** | | | **Response** | | | | | **Comments** |  |  |
| **Bias arising from the randomization process** | | 1.1 Was the allocation sequence random? | | | Y | | | | | The randomization list included a computer-generated random sequence which was implemented by the first author. |  |  |
|  |  | 1.2 Was the allocation sequence concealed until participants were enrolled and assigned to interventions? | | | PY | | | | |  |  |  |
|  |  | 1.3 Did baseline differences between intervention groups suggest a problem with the randomization process? | | | N | | | | | No imbalances are apparent or if any observed imbalances are compatible with chance. |  |  |
|  |  | **Risk of bias judgement** | | | **Low** | | | | | RoB 2 generates itself based on each of the above judgments. |  |  |
| **Bias due to deviations from intended interventions** | | 2.1.Were participants aware of their assigned intervention during the trial? | | | N | | | | | Single-blind (participants were unaware of group assignment) design.  Carers or people delivering the interventions knew to be specific to one of the interventions. |  |  |
|  |  | 2.2.Were carers and people delivering the interventions aware of participants' assigned intervention during the trial? | | | Y | | | | |  |  |  |
|  |  | 2.3. If Y/PY/NI to 2.1 or 2.2: Were there deviations from the intended intervention that arose because of the experimental context? | | | N | | | | | Changes to intervention are consistent with the trial protocol |  |  |
|  |  | 2.4 If Y/PY to 2.3: Were these deviations likely to have affected the outcome? | | | NA | | | | | Not applicable |  |  |
|  |  | 2.5. If Y/PY/NI to 2.4: Were these deviations from intended intervention balanced between groups? | | | NA | | | | | Not applicable |  |  |
|  |  | 2.6 Was an appropriate analysis used to estimate the effect of assignment to intervention? | | | Y | | | | | Postrandomization exclusions of ineligible participants can be considered appropriate. |  |  |
|  |  | 2.7 If N/PN/NI to 2.6: Was there potential for a substantial impact (on the result) of the failure to analyse participants in the group to which they were randomized? | | | NA | | | | | Not applicable |  |  |
|  |  | **Risk of bias judgement** | | | **Low** | | | | | RoB 2 generates itself based on each of the above judgments. |  |  |
| **Bias due to missing outcome data** | | 3.1 Were data for this outcome available for all, or nearly all, participants randomized? | | | PY | | | | | The appropriate study population for an analysis of the effect of LFN is all randomized participants. |  |  |
|  |  | 3.2 If N/PN/NI to 3.1: Is there evidence that result was not biased by missing outcome data? | | | NA | | | | | Not applicable |  |  |
|  |  | 3.3 If N/PN to 3.2: Could missingness in the outcome depend on its true value? | | | NA | | | | | Not applicable |  |  |
|  |  | 3.4 If Y/PY/NI to 3.3: Is it likely that missingness in the outcome depended on its true value? | | | NA | | | | | Not applicable |  |  |
|  |  | **Risk of bias judgement** | | | **Low** | | | | | RoB 2 generates itself based on each of the above judgments. |  |  |
| **Bias in measurement of the outcome** | | 4.1 Was the method of measuring the outcome inappropriate? | | | N | | | | | For pre-specified outcomes, the answer to this question will be ‘No’ or ‘Probably no. |  |  |
|  |  | 4.2 Could measurement or ascertainment of the outcome have differed between intervention groups? | | | N | | | | | Having used the same measurement. |  |  |
|  |  | 4.3 Were outcome assessors aware of the intervention received by study participants? | | | Y | | | | | Outcome assessors were aware of the intervention status. |  |  |
|  |  | 4.4 If Y/PY/NI to 4.3: Could assessment of the outcome have been influenced by knowledge of intervention received? | | | PN | | | | | The results are unlikely to influence observer-reported outcomes that do not involve judgement. |  |  |
|  |  | 4.5 If Y/PY/NI to 4.4: Is it likely that assessment of the outcome was influenced by knowledge of intervention received? | | | NA | | | | | Not applicable |  |  |
|  |  | **Risk of bias judgement** | | | **Low** | | | | | RoB 2 generates itself based on each of the above judgments. |  |  |
| **Bias in selection of the reported result** | | 5.1 Were the data that produced this result analysed in accordance with a pre-specified analysis plan that was finalized before unblinded outcome data were available for analysis? | | | Y | | | | | The researchers’ pre-specified intentions are available in sufficient detail, then planned outcome measurements and analyses can be compared with those presented in the published report(s). Trial identifier: NCT03459183, |  |  |
|  |  | 5.2 ... multiple eligible outcome measurements (e.g. scales, definitions, time points) within the outcome domain? | | | N | | | | | There is clear evidence (usually through examination of a trial protocol or statistical analysis plan) that all eligible reported results for the outcome domain correspond to all intended outcome measurements. |  |  |
|  |  | 5.3 ... multiple eligible analyses of the data? | | | N | | | | |  |  |  |
|  |  | **Risk of bias judgement** | | | **Low** | | | | | RoB 2 generates itself based on each of the above judgments. |  |  |
| **Overall bias** | | **Risk of bias judgement** | | | **Low** | | | | | RoB 2 generates itself based on the judgment of each domain above. |  |  |
|  | |  | |  | | |  | |  |  |  |  |
|  | |  | |  | | |  | |  |  |  |  |
| **Belojević G, Ohrström E, Rylander R. Effects of noise on mental performance with regard to subjective noise sensitivity. Int Arch Occup Environ Health. 1992;64(4):293-301. doi: 10.1007/BF00378288. PMID: 1468799. (Study 3)** | | | | | | | | | | |  |  |
| **Domain** | | **Signalling question** | | | **Response** | | | | | **Comments** |  |  |
| **Bias arising from the randomization process** | | 1.1 Was the allocation sequence random? | | | NI | | | | | The only information about randomization methods is a statement that the study is randomized. |  |  |
|  |  | 1.2 Was the allocation sequence concealed until participants were enrolled and assigned to interventions? | | | NI | | | | |  |  |  |
|  |  | 1.3 Did baseline differences between intervention groups suggest a problem with the randomization process? | | | PN | | | | | There is no useful baseline information available. |  |  |
|  |  | **Risk of bias judgement** | | | **Some concerns** | | | | | RoB 2 generates itself based on each of the above judgments. |  |  |
| **Bias due to deviations from intended interventions** | | 2.1.Were participants aware of their assigned intervention during the trial? | | | PY | | | | | Participants experienced some effects that they knew to be exposed to LFN. |  |  |
|  |  | 2.2.Were carers and people delivering the interventions aware of participants' assigned intervention during the trial? | | | PY | | | | | Carers or people delivering the interventions probably knew to be specific to one of the interventions. |  |  |
|  |  | 2.3. If Y/PY/NI to 2.1 or 2.2: Were there deviations from the intended intervention that arose because of the experimental context? | | | N | | | | | Changes to intervention are consistent with the trial protocol |  |  |
|  |  | 2.4 If Y/PY to 2.3: Were these deviations likely to have affected the outcome? | | | NA | | | | | Not applicable |  |  |
|  |  | 2.5. If Y/PY/NI to 2.4: Were these deviations from intended intervention balanced between groups? | | | NA | | | | | Not applicable |  |  |
|  |  | 2.6 Was an appropriate analysis used to estimate the effect of assignment to intervention? | | | PY | | | | | Postrandomization exclusions of ineligible participants can be considered appropriate. |  |  |
|  |  | 2.7 If N/PN/NI to 2.6: Was there potential for a substantial impact (on the result) of the failure to analyse participants in the group to which they were randomized? | | | NA | | | | | Not applicable |  |  |
|  |  | **Risk of bias judgement** | | | **Low** | | | | | RoB 2 generates itself based on each of the above judgments. |  |  |
| **Bias due to missing outcome data** | | 3.1 Were data for this outcome available for all, or nearly all, participants randomized? | | | Y | | | | | The appropriate study population for an analysis of the effect of LFN is all randomized participants. |  |  |
|  |  | 3.2 If N/PN/NI to 3.1: Is there evidence that result was not biased by missing outcome data? | | | NA | | | | | Not applicable |  |  |
|  |  | 3.3 If N/PN to 3.2: Could missingness in the outcome depend on its true value? | | | NA | | | | | Not applicable |  |  |
|  |  | 3.4 If Y/PY/NI to 3.3: Is it likely that missingness in the outcome depended on its true value? | | | NA | | | | | Not applicable |  |  |
|  |  | **Risk of bias judgement** | | | **Low** | | | | | RoB 2 generates itself based on each of the above judgments. |  |  |
| **Bias in measurement of the outcome** | | 4.1 Was the method of measuring the outcome inappropriate? | | | N | | | | | For pre-specified outcomes, the answer to this question will be ‘No’ or ‘Probably no. |  |  |
|  |  | 4.2 Could measurement or ascertainment of the outcome have differed between intervention groups? | | | N | | | | | Having used the same measurement. |  |  |
|  |  | 4.3 Were outcome assessors aware of the intervention received by study participants? | | | Y | | | | | Outcome assessors were aware of the intervention status. |  |  |
|  |  | 4.4 If Y/PY/NI to 4.3: Could assessment of the outcome have been influenced by knowledge of intervention received? | | | NI | | | | | Not enough information mentioned in the article. |  |  |
|  |  | 4.5 If Y/PY/NI to 4.4: Is it likely that assessment of the outcome was influenced by knowledge of intervention received? | | | PN | | | | | The results are unlikely to influence observer-reported outcomes that do not involve judgement. |  |  |
|  |  | **Risk of bias judgement** | | | **Some concerns** | | | | | RoB 2 generates itself based on each of the above judgments. |  |  |
| **Bias in selection of the reported result** | | 5.1 Were the data that produced this result analysed in accordance with a pre-specified analysis plan that was finalized before unblinded outcome data were available for analysis? | | | NI | | | | | The researchers’ pre-specified intentions are not available in detail. |  |  |
|  |  | 5.2 ... multiple eligible outcome measurements (e.g. scales, definitions, time points) within the outcome domain? | | | PN | | | | | There is clear evidence (usually through examination of a trial protocol or statistical analysis plan) that all eligible reported results for the outcome domain correspond to all intended outcome measurements. |  |  |
|  |  | 5.3 ... multiple eligible analyses of the data? | | | PN | | | | |  |  |  |
|  |  | **Risk of bias judgement** | | | **Some concerns** | | | | | RoB 2 generates itself based on each of the above judgments. |  |  |
| **Overall bias** | | **Risk of bias judgement** | | | **Some concerns** | | | | | RoB 2 generates itself based on the judgment of each domain above. |  |  |
|  | |  | |  | | |  | |  |  |  |  |
|  | |  | |  | | |  | |  |  |  |  |
| **Ljung R, Sörqvist P, Hygge S. Effects of road traffic noise and irrelevant speech on children's reading and mathematical performance. Noise Health. 2009 Oct-Dec;11(45):194-8. doi: 10.4103/1463-1741.56212. PMID: 19805928. (Study 4)** | | | | | | | | | | |  |  |
| **Domain** | | **Signalling question** | | | **Response** | | | | | **Comments** |  |  |
| **Bias arising from the randomization process** | | 1.1 Was the allocation sequence random? | | | NI | | | | | The only information about randomization methods is a statement that the study is randomized. |  |  |
|  |  | 1.2 Was the allocation sequence concealed until participants were enrolled and assigned to interventions? | | | NI | | | | |  |  |  |
|  |  | 1.3 Did baseline differences between intervention groups suggest a problem with the randomization process? | | | PN | | | | | There is no useful baseline information available. |  |  |
|  |  | **Risk of bias judgement** | | | **Some concerns** | | | | | RoB 2 generates itself based on each of the above judgments. |  |  |
| **Bias due to deviations from intended interventions** | | 2.1.Were participants aware of their assigned intervention during the trial? | | | PY | | | | | Participants experienced some effects that they knew to be exposed to LFN. |  |  |
|  |  | 2.2.Were carers and people delivering the interventions aware of participants' assigned intervention during the trial? | | | PY | | | | | Carers or people delivering the interventions probably knew to be specific to one of the interventions. |  |  |
|  |  | 2.3. If Y/PY/NI to 2.1 or 2.2: Were there deviations from the intended intervention that arose because of the experimental context? | | | PN | | | | | Changes to intervention are consistent with the trial protocol |  |  |
|  |  | 2.4 If Y/PY to 2.3: Were these deviations likely to have affected the outcome? | | | NA | | | | | Not applicable |  |  |
|  |  | 2.5. If Y/PY/NI to 2.4: Were these deviations from intended intervention balanced between groups? | | | NA | | | | | Not applicable |  |  |
|  |  | 2.6 Was an appropriate analysis used to estimate the effect of assignment to intervention? | | | PY | | | | | Postrandomization exclusions of ineligible participants can be considered appropriate. |  |  |
|  |  | 2.7 If N/PN/NI to 2.6: Was there potential for a substantial impact (on the result) of the failure to analyse participants in the group to which they were randomized? | | | NA | | | | | Not applicable |  |  |
|  |  | **Risk of bias judgement** | | | **Low** | | | | | RoB 2 generates itself based on each of the above judgments. |  |  |
| **Bias due to missing outcome data** | | 3.1 Were data for this outcome available for all, or nearly all, participants randomized? | | | PY | | | | | The appropriate study population for an analysis of the effect of LFN is all randomized participants. |  |  |
|  |  | 3.2 If N/PN/NI to 3.1: Is there evidence that result was not biased by missing outcome data? | | | NA | | | | | Not applicable |  |  |
|  |  | 3.3 If N/PN to 3.2: Could missingness in the outcome depend on its true value? | | | NA | | | | | Not applicable |  |  |
|  |  | 3.4 If Y/PY/NI to 3.3: Is it likely that missingness in the outcome depended on its true value? | | | NA | | | | | Not applicable |  |  |
|  |  | **Risk of bias judgement** | | | **Low** | | | | | RoB 2 generates itself based on each of the above judgments. |  |  |
| **Bias in measurement of the outcome** | | 4.1 Was the method of measuring the outcome inappropriate? | | | PN | | | | | For pre-specified outcomes, the answer to this question will be ‘No’ or ‘Probably no. |  |  |
|  |  | 4.2 Could measurement or ascertainment of the outcome have differed between intervention groups? | | | N | | | | | Having used the same measurement. |  |  |
|  |  | 4.3 Were outcome assessors aware of the intervention received by study participants? | | | PY | | | | | Outcome assessors were aware of the intervention status. |  |  |
|  |  | 4.4 If Y/PY/NI to 4.3: Could assessment of the outcome have been influenced by knowledge of intervention received? | | | PN | | | | | The results are unlikely to influence observer-reported outcomes that do not involve judgement. |  |  |
|  |  | 4.5 If Y/PY/NI to 4.4: Is it likely that assessment of the outcome was influenced by knowledge of intervention received? | | | NA | | | | | Not applicable |  |  |
|  |  | **Risk of bias judgement** | | | **Low** | | | | | RoB 2 generates itself based on each of the above judgments. |  |  |
| **Bias in selection of the reported result** | | 5.1 Were the data that produced this result analysed in accordance with a pre-specified analysis plan that was finalized before unblinded outcome data were available for analysis? | | | NI | | | | | The researchers’ pre-specified intentions are not available in detail. |  |  |
|  |  | 5.2 ... multiple eligible outcome measurements (e.g. scales, definitions, time points) within the outcome domain? | | | PN | | | | | There is clear evidence (usually through examination of a trial protocol or statistical analysis plan) that all eligible reported results for the outcome domain correspond to all intended outcome measurements. |  |  |
|  |  | 5.3 ... multiple eligible analyses of the data? | | | PN | | | | |  |  |  |
|  |  | **Risk of bias judgement** | | | **Some concerns** | | | | | RoB 2 generates itself based on each of the above judgments. |  |  |
| **Overall bias** | | **Risk of bias judgement** | | | **Some concerns** | | | | | RoB 2 generates itself based on the judgment of each domain above. |  |  |
|  | |  | |  | | |  | |  |  |  |  |
|  | |  | |  | | |  | |  |  |  |  |
| **Persson Waye K, Bengtsson J, Kjellberg A, Benton S. Low frequency noise "pollution" interferes with performance. Noise Health. 2001;4(13):33-49. PMID: 12678934. (Study 5)** | | | | | | | | | | |  |  |
| **Domain** | | **Signalling question** | | | **Response** | | | | | **Comments** |  |  |
| **Bias arising from the randomization process** | | 1.1 Was the allocation sequence random? | | | NI | | | | | The only information about randomization methods is a statement that the study is randomized. |  |  |
|  |  | 1.2 Was the allocation sequence concealed until participants were enrolled and assigned to interventions? | | | NI | | | | |  |  |  |
|  |  | 1.3 Did baseline differences between intervention groups suggest a problem with the randomization process? | | | PN | | | | | There is no useful baseline information available. |  |  |
|  |  | **Risk of bias judgement** | | | **Some concerns** | | | | | RoB 2 generates itself based on each of the above judgments. |  |  |
| **Bias due to deviations from intended interventions** | | 2.1.Were participants aware of their assigned intervention during the trial? | | | PY | | | | | Participants experienced some effects that they knew to be exposed to LFN. |  |  |
|  |  | 2.2.Were carers and people delivering the interventions aware of participants' assigned intervention during the trial? | | | PY | | | | | Carers or people delivering the interventions probably knew to be specific to one of the interventions. |  |  |
|  |  | 2.3. If Y/PY/NI to 2.1 or 2.2: Were there deviations from the intended intervention that arose because of the experimental context? | | | PN | | | | | Changes to intervention are consistent with the trial protocol |  |  |
|  |  | 2.4 If Y/PY to 2.3: Were these deviations likely to have affected the outcome? | | | NA | | | | | Not applicable |  |  |
|  |  | 2.5. If Y/PY/NI to 2.4: Were these deviations from intended intervention balanced between groups? | | | NA | | | | | Not applicable |  |  |
|  |  | 2.6 Was an appropriate analysis used to estimate the effect of assignment to intervention? | | | Y | | | | | Postrandomization exclusions of ineligible participants can be considered appropriate. |  |  |
|  |  | 2.7 If N/PN/NI to 2.6: Was there potential for a substantial impact (on the result) of the failure to analyse participants in the group to which they were randomized? | | | NA | | | | | Not applicable |  |  |
|  |  | **Risk of bias judgement** | | | **Low** | | | | | RoB 2 generates itself based on each of the above judgments. |  |  |
| **Bias due to missing outcome data** | | 3.1 Were data for this outcome available for all, or nearly all, participants randomized? | | | PY | | | | | The appropriate study population for an analysis of the effect of LFN is all randomized participants. |  |  |
|  |  | 3.2 If N/PN/NI to 3.1: Is there evidence that result was not biased by missing outcome data? | | | NA | | | | | Not applicable |  |  |
|  |  | 3.3 If N/PN to 3.2: Could missingness in the outcome depend on its true value? | | | NA | | | | | Not applicable |  |  |
|  |  | 3.4 If Y/PY/NI to 3.3: Is it likely that missingness in the outcome depended on its true value? | | | NA | | | | | Not applicable |  |  |
|  |  | **Risk of bias judgement** | | | **Low** | | | | | RoB 2 generates itself based on each of the above judgments. |  |  |
| **Bias in measurement of the outcome** | | 4.1 Was the method of measuring the outcome inappropriate? | | | PN | | | | | For pre-specified outcomes, the answer to this question will be ‘No’ or ‘Probably no. |  |  |
|  |  | 4.2 Could measurement or ascertainment of the outcome have differed between intervention groups? | | | N | | | | | Having used the same measurement. |  |  |
|  |  | 4.3 Were outcome assessors aware of the intervention received by study participants? | | | PY | | | | | Outcome assessors were aware of the intervention status. |  |  |
|  |  | 4.4 If Y/PY/NI to 4.3: Could assessment of the outcome have been influenced by knowledge of intervention received? | | | PN | | | | | The results are unlikely to influence observer-reported outcomes that do not involve judgement. |  |  |
|  |  | 4.5 If Y/PY/NI to 4.4: Is it likely that assessment of the outcome was influenced by knowledge of intervention received? | | | NA | | | | | Not applicable |  |  |
|  |  | **Risk of bias judgement** | | | **Low** | | | | | RoB 2 generates itself based on each of the above judgments. |  |  |
| **Bias in selection of the reported result** | | 5.1 Were the data that produced this result analysed in accordance with a pre-specified analysis plan that was finalized before unblinded outcome data were available for analysis? | | | NI | | | | | The researchers’ pre-specified intentions are not available in detail. |  |  |
|  |  | 5.2 ... multiple eligible outcome measurements (e.g. scales, definitions, time points) within the outcome domain? | | | N | | | | | There is clear evidence (usually through examination of a trial protocol or statistical analysis plan) that all eligible reported results for the outcome domain correspond to all intended outcome measurements. |  |  |
|  |  | 5.3 ... multiple eligible analyses of the data? | | | N | | | | |  |  |  |
|  |  | **Risk of bias judgement** | | | **Some concerns** | | | | | RoB 2 generates itself based on each of the above judgments. |  |  |
| **Overall bias** | | **Risk of bias judgement** | | | **Some concerns** | | | | | RoB 2 generates itself based on the judgment of each domain above. |  |  |
|  | |  | |  | | |  | |  |  |  |  |
|  | |  | |  | | |  | |  |  |  |  |
| **Alimohammadi I, Zokaei M, Sandrock S. The Effect of Road Traffic Noise on Reaction Time. Health Promot Perspect. 2015 Oct 25;5(3):207-14. doi: 10.15171/hpp.2015.025. PMID: 26634199; PMCID: PMC4667263. (Study 6)** | | | | | | | | | | |  |  |
| **Domain** | | **Signalling question** | | | **Response** | | | | | **Comments** |  |  |
| **Bias arising from the randomization process** | | 1.1 Was the allocation sequence random? | | | PY | | | | | Forty extraverts and forty introverts were randomly selected. Then, the participants were randomly assigned to either the case or the control group. |  |  |
|  |  | 1.2 Was the allocation sequence concealed until participants were enrolled and assigned to interventions? | | | PY | | | | |  |  |  |
|  |  | 1.3 Did baseline differences between intervention groups suggest a problem with the randomization process? | | | PN | | | | | There is no useful baseline information available. |  |  |
|  |  | **Risk of bias judgement** | | | **Low** | | | | | RoB 2 generates itself based on each of the above judgments. |  |  |
| **Bias due to deviations from intended interventions** | | 2.1.Were participants aware of their assigned intervention during the trial? | | | PY | | | | | Participants experienced some effects that they knew to be exposed to LFN. |  |  |
|  |  | 2.2.Were carers and people delivering the interventions aware of participants' assigned intervention during the trial? | | | PY | | | | | Carers or people delivering the interventions probably knew to be specific to one of the interventions. |  |  |
|  |  | 2.3. If Y/PY/NI to 2.1 or 2.2: Were there deviations from the intended intervention that arose because of the experimental context? | | | PN | | | | | Changes to intervention are consistent with the trial protocol |  |  |
|  |  | 2.4 If Y/PY to 2.3: Were these deviations likely to have affected the outcome? | | | NA | | | | | Not applicable |  |  |
|  |  | 2.5. If Y/PY/NI to 2.4: Were these deviations from intended intervention balanced between groups? | | | NA | | | | | Not applicable |  |  |
|  |  | 2.6 Was an appropriate analysis used to estimate the effect of assignment to intervention? | | | PY | | | | | Postrandomization exclusions of ineligible participants can be considered appropriate. |  |  |
|  |  | 2.7 If N/PN/NI to 2.6: Was there potential for a substantial impact (on the result) of the failure to analyse participants in the group to which they were randomized? | | | NA | | | | | Not applicable |  |  |
|  |  | **Risk of bias judgement** | | | **Low** | | | | | RoB 2 generates itself based on each of the above judgments. |  |  |
| **Bias due to missing outcome data** | | 3.1 Were data for this outcome available for all, or nearly all, participants randomized? | | | Y | | | | | The appropriate study population for an analysis of the effect of LFN is all randomized participants. |  |  |
|  |  | 3.2 If N/PN/NI to 3.1: Is there evidence that result was not biased by missing outcome data? | | | NA | | | | | Not applicable |  |  |
|  |  | 3.3 If N/PN to 3.2: Could missingness in the outcome depend on its true value? | | | NA | | | | | Not applicable |  |  |
|  |  | 3.4 If Y/PY/NI to 3.3: Is it likely that missingness in the outcome depended on its true value? | | | NA | | | | | Not applicable |  |  |
|  |  | **Risk of bias judgement** | | | **Low** | | | | | RoB 2 generates itself based on each of the above judgments. |  |  |
| **Bias in measurement of the outcome** | | 4.1 Was the method of measuring the outcome inappropriate? | | | N | | | | | For pre-specified outcomes, the answer to this question will be ‘No’ or ‘Probably no. |  |  |
|  |  | 4.2 Could measurement or ascertainment of the outcome have differed between intervention groups? | | | N | | | | | Having used the same measurement. |  |  |
|  |  | 4.3 Were outcome assessors aware of the intervention received by study participants? | | | PY | | | | | Outcome assessors were aware of the intervention status. |  |  |
|  |  | 4.4 If Y/PY/NI to 4.3: Could assessment of the outcome have been influenced by knowledge of intervention received? | | | PN | | | | | The results are unlikely to influence observer-reported outcomes that do not involve judgement. |  |  |
|  |  | 4.5 If Y/PY/NI to 4.4: Is it likely that assessment of the outcome was influenced by knowledge of intervention received? | | | NA | | | | | Not applicable |  |  |
|  |  | **Risk of bias judgement** | | | **Low** | | | | | RoB 2 generates itself based on each of the above judgments. |  |  |
| **Bias in selection of the reported result** | | 5.1 Were the data that produced this result analysed in accordance with a pre-specified analysis plan that was finalized before unblinded outcome data were available for analysis? | | | PY | | | | | The researchers’ pre-specified intentions are available in sufficient detail, then planned outcome measurements and analyses can be compared with those presented in the published report(s). |  |  |
|  |  | 5.2 ... multiple eligible outcome measurements (e.g. scales, definitions, time points) within the outcome domain? | | | N | | | | | There is clear evidence (usually through examination of a trial protocol or statistical analysis plan) that all eligible reported results for the outcome domain correspond to all intended outcome measurements. |  |  |
|  |  | 5.3 ... multiple eligible analyses of the data? | | | N | | | | |  |  |  |
|  |  | **Risk of bias judgement** | | | **Low** | | | | | RoB 2 generates itself based on each of the above judgments. |  |  |
| **Overall bias** | | **Risk of bias judgement** | | | **Low** | | | | | RoB 2 generates itself based on the judgment of each domain above. |  |  |
|  | |  | |  | | |  | |  |  |  |  |
|  | |  | |  | | |  | |  |  |  |  |
| **Alimohammadi I, Sandrock S, Gohari MR. The effects of low frequency noise on mental performance and annoyance. Environ Monit Assess. 2013 Aug;185(8):7043-51. doi: 10.1007/s10661-013-3084-8. Epub 2013 Jan 22. PMID: 23338951. (Study 7)** | | | | | | | | | | |  |  |
| **Domain** | | **Signalling question** | | | **Response** | | | | | **Comments** |  |  |
| **Bias arising from the randomization process** | 1.1 Was the allocation sequence random? | | | | PN | | | | | Absence of specific information about generation of the randomization sequence. No random element was used in generating the allocation sequence. |  |  |
|  | 1.2 Was the allocation sequence concealed until participants were enrolled and assigned to interventions? | | | | NI | | | | |  |  |  |
|  | 1.3 Did baseline differences between intervention groups suggest a problem with the randomization process? | | | | PN | | | | | There is no useful baseline information available. |  |  |
|  | **Risk of bias judgement** | | | | **Some concerns** | | | | | RoB 2 generates itself based on each of the above judgments. |  |  |
| **Bias due to deviations from intended interventions** | 2.1.Were participants aware of their assigned intervention during the trial? | | | | PY | | | | | Participants experienced some effects that they knew to be exposed to LFN. |  |  |
|  | 2.2.Were carers and people delivering the interventions aware of participants' assigned intervention during the trial? | | | | PY | | | | | Carers or people delivering the interventions probably knew to be specific to one of the interventions. |  |  |
|  | 2.3. If Y/PY/NI to 2.1 or 2.2: Were there deviations from the intended intervention that arose because of the experimental context? | | | | PN | | | | | Changes to intervention are consistent with the trial protocol |  |  |
|  | 2.4 If Y/PY to 2.3: Were these deviations likely to have affected the outcome? | | | | NA | | | | | Not applicable |  |  |
|  | 2.5. If Y/PY/NI to 2.4: Were these deviations from intended intervention balanced between groups? | | | | NA | | | | | Not applicable |  |  |
|  | 2.6 Was an appropriate analysis used to estimate the effect of assignment to intervention? | | | | PY | | | | | Postrandomization exclusions of ineligible participants can be considered appropriate. |  |  |
|  | 2.7 If N/PN/NI to 2.6: Was there potential for a substantial impact (on the result) of the failure to analyse participants in the group to which they were randomized? | | | | NA | | | | | Not applicable |  |  |
|  | **Risk of bias judgement** | | | | **Low** | | | | | RoB 2 generates itself based on each of the above judgments. |  |  |
| **Bias due to missing outcome data** | 3.1 Were data for this outcome available for all, or nearly all, participants randomized? | | | | PY | | | | | The appropriate study population for an analysis of the effect of LFN is all randomized participants. |  |  |
|  | 3.2 If N/PN/NI to 3.1: Is there evidence that result was not biased by missing outcome data? | | | | NA | | | | | Not applicable |  |  |
|  | 3.3 If N/PN to 3.2: Could missingness in the outcome depend on its true value? | | | | NA | | | | | Not applicable |  |  |
|  | 3.4 If Y/PY/NI to 3.3: Is it likely that missingness in the outcome depended on its true value? | | | | NA | | | | | Not applicable |  |  |
|  | **Risk of bias judgement** | | | | **Low** | | | | | RoB 2 generates itself based on each of the above judgments. |  |  |
| **Bias in measurement of the outcome** | 4.1 Was the method of measuring the outcome inappropriate? | | | | PN | | | | | For pre-specified outcomes, the answer to this question will be ‘No’ or ‘Probably no. |  |  |
|  | 4.2 Could measurement or ascertainment of the outcome have differed between intervention groups? | | | | PN | | | | | Having used the same measurement. |  |  |
|  | 4.3 Were outcome assessors aware of the intervention received by study participants? | | | | PY | | | | | Outcome assessors were aware of the intervention status. |  |  |
|  | 4.4 If Y/PY/NI to 4.3: Could assessment of the outcome have been influenced by knowledge of intervention received? | | | | PN | | | | | The results are unlikely to influence observer-reported outcomes that do not involve judgement. |  |  |
|  | 4.5 If Y/PY/NI to 4.4: Is it likely that assessment of the outcome was influenced by knowledge of intervention received? | | | | NA | | | | | Not applicable |  |  |
|  | **Risk of bias judgement** | | | | **Low** | | | | | RoB 2 generates itself based on each of the above judgments. |  |  |
| **Bias in selection of the reported result** | 5.1 Were the data that produced this result analysed in accordance with a pre-specified analysis plan that was finalized before unblinded outcome data were available for analysis? | | | | NI | | | | | The researchers’ pre-specified intentions are not available in detail. |  |  |
|  | 5.2 ... multiple eligible outcome measurements (e.g. scales, definitions, time points) within the outcome domain? | | | | PN | | | | | There is clear evidence (usually through examination of a trial protocol or statistical analysis plan) that all eligible reported results for the outcome domain correspond to all intended outcome measurements. |  |  |
|  | 5.3 ... multiple eligible analyses of the data? | | | | PN | | | | |  |  |  |
|  | **Risk of bias judgement** | | | | **Some concerns** | | | | | RoB 2 generates itself based on each of the above judgments. |  |  |
| **Overall bias** | **Risk of bias judgement** | | | | **Some concerns** | | | | | RoB 2 generates itself based on the judgment of each domain above. |  |  |
|  |  | |  | | |  | |  | |  |  |  |
|  |  | |  | | |  | |  | |  |  |  |
| **Pawlaczyk-Luszczyńiska M, Dudarewicz A, Waszkowska M, Szymczak W, Sliwińska-Kowalska M. The impact of low-frequency noise on human mental performance. Int J Occup Med Environ Health. 2005;18(2):185-98. PMID: 16201210. (Study 8)** | | | | | | | | | | |  |  |
| **Domain** | **Signalling question** | | | | **Response** | | | | | **Comments** |  |  |
| **Bias arising from the randomization process** | 1.1 Was the allocation sequence random? | | | | NI | | | | | The only information about randomization methods is a statement that the study is randomized. The sequence was concealed before. |  |  |
|  | 1.2 Was the allocation sequence concealed until participants were enrolled and assigned to interventions? | | | | PY | | | | |  |  |  |
|  | 1.3 Did baseline differences between intervention groups suggest a problem with the randomization process? | | | | PN | | | | | There is no useful baseline information available. |  |  |
|  | **Risk of bias judgement** | | | | **Low** | | | | | RoB 2 generates itself based on each of the above judgments. |  |  |
| **Bias due to deviations from intended interventions** | 2.1.Were participants aware of their assigned intervention during the trial? | | | | Y | | | | | Participants experienced some effects that they knew to be exposed to LFN. |  |  |
|  | 2.2.Were carers and people delivering the interventions aware of participants' assigned intervention during the trial? | | | | PY | | | | | Carers or people delivering the interventions probably knew to be specific to one of the interventions. |  |  |
|  | 2.3. If Y/PY/NI to 2.1 or 2.2: Were there deviations from the intended intervention that arose because of the experimental context? | | | | PN | | | | | Changes to intervention are consistent with the trial protocol |  |  |
|  | 2.4 If Y/PY to 2.3: Were these deviations likely to have affected the outcome? | | | | NA | | | | | Not applicable |  |  |
|  | 2.5. If Y/PY/NI to 2.4: Were these deviations from intended intervention balanced between groups? | | | | NA | | | | | Not applicable |  |  |
|  | 2.6 Was an appropriate analysis used to estimate the effect of assignment to intervention? | | | | PY | | | | | Postrandomization exclusions of ineligible participants can be considered appropriate. |  |  |
|  | 2.7 If N/PN/NI to 2.6: Was there potential for a substantial impact (on the result) of the failure to analyse participants in the group to which they were randomized? | | | | NA | | | | | Not applicable |  |  |
|  | **Risk of bias judgement** | | | | **Low** | | | | | RoB 2 generates itself based on each of the above judgments. |  |  |
| **Bias due to missing outcome data** | 3.1 Were data for this outcome available for all, or nearly all, participants randomized? | | | | Y | | | | | The appropriate study population for an analysis of the effect of LFN is all randomized participants. |  |  |
|  | 3.2 If N/PN/NI to 3.1: Is there evidence that result was not biased by missing outcome data? | | | | NA | | | | | Not applicable |  |  |
|  | 3.3 If N/PN to 3.2: Could missingness in the outcome depend on its true value? | | | | NA | | | | | Not applicable |  |  |
|  | 3.4 If Y/PY/NI to 3.3: Is it likely that missingness in the outcome depended on its true value? | | | | NA | | | | | Not applicable |  |  |
|  | **Risk of bias judgement** | | | | **Low** | | | | | RoB 2 generates itself based on each of the above judgments. |  |  |
| **Bias in measurement of the outcome** | 4.1 Was the method of measuring the outcome inappropriate? | | | | PN | | | | | For pre-specified outcomes, the answer to this question will be ‘No’ or ‘Probably no. |  |  |
|  | 4.2 Could measurement or ascertainment of the outcome have differed between intervention groups? | | | | N | | | | | Having used the same measurement. |  |  |
|  | 4.3 Were outcome assessors aware of the intervention received by study participants? | | | | PY | | | | | Outcome assessors were aware of the intervention status. |  |  |
|  | 4.4 If Y/PY/NI to 4.3: Could assessment of the outcome have been influenced by knowledge of intervention received? | | | | PN | | | | | The results are unlikely to influence observer-reported outcomes that do not involve judgement. |  |  |
|  | 4.5 If Y/PY/NI to 4.4: Is it likely that assessment of the outcome was influenced by knowledge of intervention received? | | | | NA | | | | | Not applicable |  |  |
|  | **Risk of bias judgement** | | | | **Low** | | | | | RoB 2 generates itself based on each of the above judgments. |  |  |
| **Bias in selection of the reported result** | 5.1 Were the data that produced this result analysed in accordance with a pre-specified analysis plan that was finalized before unblinded outcome data were available for analysis? | | | | NI | | | | | The researchers’ pre-specified intentions are not available in detail. |  |  |
|  | 5.2 ... multiple eligible outcome measurements (e.g. scales, definitions, time points) within the outcome domain? | | | | PN | | | | | There is clear evidence (usually through examination of a trial protocol or statistical analysis plan) that all eligible reported results for the outcome domain correspond to all intended outcome measurements. |  |  |
|  | 5.3 ... multiple eligible analyses of the data? | | | | PN | | | | |  |  |  |
|  | **Risk of bias judgement** | | | | **Some concerns** | | | | | RoB 2 generates itself based on each of the above judgments. |  |  |
| **Overall bias** | **Risk of bias judgement** | | | | **Some concerns** | | | | | RoB 2 generates itself based on the judgment of each domain above. |  |  |

**Y/PY = ‘Yes’ or ‘Probably yes’; N/PN = ‘No’ or ‘Probably no’; NI = ‘No information’; NA = Not applicable**
