# Supplementary material for: Effect of low-frequency noise exposure on cognitive function: a systematic review and meta-analysis
Source: BMC Public Health. 2024 Jan 9;24:125. doi: 10.1186/s12889-023-17593-5 (PMC10775542; doi:10.1186/s12889-023-17593-5)
Supplement: Supplementary file 6 — Additional file 6. GRADE summary for quality of evidence from LFN associated with cognitive function. [file 12889_2023_17593_MOESM6_ESM.docx]

**Additional file 6. GRADE summary for quality of evidence from LFN associated with cognitive function.**

| **Quality assessment** | | | | | | | **No of patients** | | **Effect** | | **Quality** | **Importance** |  |
| --- | --- | --- | --- | --- | --- | --- | --- | --- | --- | --- | --- | --- | --- |
|  |  |  |  |  |  |  |  |  |  |  |  |  |  |
| **No of studies** | **Design** | **Risk of bias** | **Inconsistency** | **Indirectness** | **Imprecision** | **Other considerations** | **LFN** | **Control** | **Relative (95% CI)** | **Absolute** |  |  |  |
| **Attentional functioning** | | | | | | | | | | | | |  |
| 5 | randomised trials | serious^1^ | very serious^2^ | no serious indirectness | serious^3^ | none | 213 | 205 | - | SMD 0.05 lower  (0.75 lower to 0.66 higher) | ⊕OOO **VERY LOW** | CRITICAL |  |
| **Executive functioning** | | | | | | | | | | | | |  |
| 7 | randomised trials | serious^1^ | no serious inconsistency^4^ | no serious indirectness | serious^3^ | none | 308 | 320 | - | SMD 0.06 lower  (0.22 lower to 0.1 higher) | ⊕⊕OO **LOW** | CRITICAL |  |
| **Memory** | | | | | | | | | | | | |  |
| 4 | randomised trials | serious^1^ | no serious inconsistency^5^ | no serious indirectness | very serious^6^ | none | 98 | 88 | - | SMD 0.09 lower  (0.38 lower to 0.2 higher) | ⊕OOO **VERY LOW** | CRITICAL |  |
| **Higher-order functions** | | | | | | | | | | | | |  |
| 4 | randomised trials | serious^1^ | no serious inconsistency^7^ | no serious indirectness | serious^8^ | none | 133 | 145 | - | SMD 0.37 lower  (0.67 to 0.07 lower) | ⊕⊕OO **LOW** | CRITICAL |  |

^1^ The only information about randomization methods is a statement that the study is randomized. Most information is from studies at low or unclear risk of bias.
^2^ There is high heterogeneity among the studies (p<0.001, I^2^=91%, Tau^2^=0.56).
^3^ The 95% confidence interval includes no effect and the upper or lower confidence limit crosses the minimal important difference (MID).
^4^ The studies exhibited no heterogeneity (p=0.58, I^2^=0%, Tau^2^=0.00)
^5^ The studies demonstrated no heterogeneity (p=0.90, I^2^=0%, Tau^2^=0.00).
^6^ The 95% confidence interval includes no effect and the upper or lower confidence limit crosses the MID and the total population size is less than 400.
^7^ The studies demonstrated moderate heterogeneity (p=0.24, I^2^=29%, Tau^2^=0.03).
^8^ The total population size is less than 400.
